# *Cyclo*‐P_5_^−^ Revisited: The Surprisingly Stable Uncoordinated Pentaphospholide Anion

**DOI:** 10.1002/anie.202505853

**Published:** 2025-04-27

**Authors:** Moritz J. Ernst, Andrey Petrov, Mirjam Schröder, Björn Corzilius, Christian Müller

**Affiliations:** ^1^ Institute of Chemistry and Biochemistry Freie Universität Berlin Fabeckstr. 34/36 14195 Berlin Germany; ^2^ Leibniz‐Institute for Catalysis Albert‐Einstein‐Str. 29a 18059 Rostock Germany; ^3^ University of Rostock, Institute of Chemistry and Department of Life, Light & Matter 18059 Rostock Germany

**Keywords:** ^31^P MAS NMR spectroscopy, Cyclopentadienyl ligands, Density functional calculations, Phosphorus cycles, X‐ray diffraction

## Abstract

The addition of [2.2.2]cryptand to alkali metal heptaphosphides M_3_P_7_ (M = Na, K) leads to the formation of [M([2.2.2]cryptand)][*cyclo*‐P_5_] salts. Although the *cyclo*‐P_5_^−^ anion is spectroscopically known since the 1980s, it was so far prepared and handled only in solution. By complexing the alkali metal cation with [2.2.2]cryptand, the product was found to be surprisingly stable in the solid state. *Cyclo*‐P_5_^−^, which is isolobal to the well‐known cyclopentadienide anion, was now characterized crystallographically for the first time in its uncoordinated form and in the presence of the weakly coordinating cations [M(2.2.2‐cryptand)]^+^ (M = Na, K). The structural elucidation proves its planar *D_5_* *_h_* symmetry, not only as ligand in different sandwich complexes but also in its uncoordinated form. C*yclo*‐P_5_^−^ was further characterized by UV/Visis spectroscopy in solution and in the solid state by Raman and ^31^P MAS NMR spectroscopy. The reaction of [Na([2.2.2]cryptand)][*cyclo*‐P_5_] with LiCp* and FeCl_2_ yields the ferrocene derivative [Cp*Fe(*cyclo*‐P_5_)].

## Graphical abstract

The intriguing c*yclo*‐P_5_^−^ anion, which is isolobal to the well‐known cyclopentadienide anion, was characterized for the first time crystallographically in the salts [M([2.2.2]cryptand)][*cyclo*‐P_5_] (M = Na, K). The compounds were further characterized by UV/Vis spectroscopy in solution and in the solid state by Raman and ^31^P MAS NMR spectroscopy.

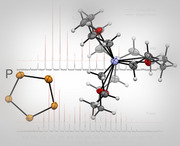

The electron‐rich, aromatic cyclopentadienide anion C_5_H_5_^−^ (**A**, Figure 1) is undoubtedly one of the most versatile ligands in organometallic and coordination chemistry and plays an important role in both fundamental research and practical applications. Taking the isolobal principle into account, the related monocyclic compounds *cyclo*‐Pn_5_^−^ (**B**–**F**, Figure 1) are known for all pnictogens (Pn). Interestingly, among the series **B**–**F**, only the *cyclo*‐N_5_^−^ (**B**) has so far been characterized crystallographically in its uncoordinated form.^[^ ^1^, ^2^, ^3^ ^]^ In the first reports on the salt (N_5_)_6_(H_3_O)_3_(NH_4_)_4_Cl by Ming Lu and co‐workers in 2017, hydrogen bonding between the ammonium and oxonium ions and the nitrogen atoms of the pentazolate anion was also observed.^[^ ^1^ ^]^

**Figure 1:**
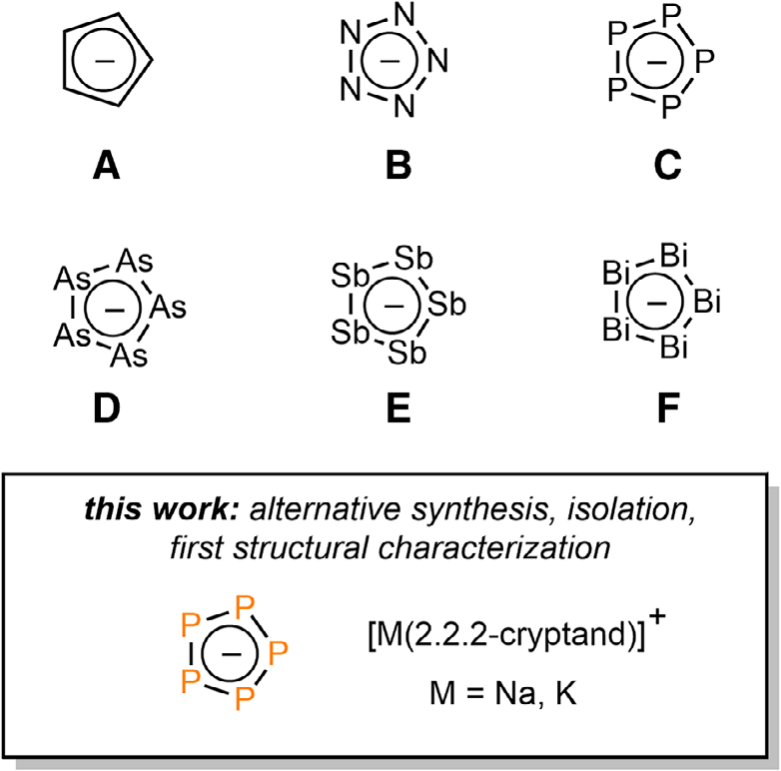
The parent cyclopentadienyl anion (**A**), the isolobal pnictogen *cyclo*‐Pn_5_^−^ anions (**B**–**F**) and a short summary of this work.

Since the discovery of *cyclo*‐P_5_^−^ by Baudler and co‐worker, it was generally accepted that **C** exists only in solution, as it oligomerizes rapidly upon removing the solvent.^[^ ^4^, ^5^, ^6^ ^]^ Nevertheless, the evidence of its aromatic and highly symmetric structure is evident from the corresponding ^31^P NMR spectrum, which shows only one significantly deshielded singlet resonance with a chemical shift at *δ*(ppm) = 470.0.^[^ ^4^ ^]^ Ab initio calculations of the *cyclo*‐P_5_^−^ anion also confirmed the *D_5_* *_h_* planar cyclic structure with a P─P bond length of around 2.093 Å.^[^ ^7^, ^8^ ^]^ Several computational studies on the electronic structure and aromaticity of *cyclo*‐P_5_^−^, especially in comparison with its isolobal analogue C_5_H_5_^−^, have been reported in the last decades.^[^ ^9^, ^10^, ^11^, ^12^, ^13^, ^14^ ^]^ Moreover, *cyclo*‐P_5_^−^ was investigated intensively as ligand in sandwich complexes.^[^ ^15^, ^16^, ^17^, ^18^, ^19^, ^20^ ^]^ The majority of such coordination compounds were synthesized by activation of white phosphorus with transition metal complexes to access, for example, the well‐known ferrocene derivative [Cp*Fe(*cyclo*‐P_5_)], the related Ru(II) complex [Cp*Ru(*cyclo*‐P_5_)], or the carbon‐free sandwich complex [Ti(*cyclo*‐P_5_)_2_]^2−^.^[^ ^16^, ^17^, ^18^, ^19^ ^]^ [Cp*Fe(*cyclo*‐P_5_)] was shown to act as *cyclo‐P_5_^−^‐*transfer agent yielding, for example, the corresponding osmium complex.^[^ ^20^ ^]^ Moreover, the generation of *cyclo*‐P_5_^−^ in solution has opened up the possibility to transfer this anionic homocycle as ligand into coordination compounds.^[^ ^21^ ^]^ The presence of energetically and sterically accessible lone pairs at each phosphorus atom further led to the synthesis of polynuclear and supramolecular coordination compounds,^[^ ^22^, ^23^, ^24^ ^]^ while investigations on their reactivity towards nucleophiles and electrophiles were impressively demonstrated by Scheer, Roesky and co‐workers.^[^ ^25^, ^26^, ^27^ ^]^

In contrast, much less is known about *cyclo*‐As_5_^−^ and *cyclo*‐Sb_5_^−^. The cyclopentaarsenide anion **D** has been generated starting from As_4_ and was isolated as both triple‐decker and sandwich‐complexes, while the pentastibacyclopentadienide anion **E** is accessible from *cyclo*‐*t*Bu_4_Sb_4_.^[^ ^28^, ^29^ ^]^ Again, **E** was so far only characterized crystallographically as a triple‐decker Mo‐sandwich complex.^[^ ^29^ ^]^ Finally, Dehnen and co‐workers recently reported on the successful generation of *cyclo*‐Bi_5_^−^ (**F**), the heaviest analogue of C_5_H_5_^−^, and its crystallographic characterization as the anionic Co‐complex [IMes_2_Co_2_Bi_5_]^−^ (IMes: bis(1,3‐(2,4,6‐trimethylphenyl))‐imidazol‐2‐ylidene).^[^ ^30^ ^]^ During our investigation on the reactivity of **C** towards C≡E (E = C, N, P) triple bonds,^[^ ^31^ ^]^ we noticed that the chemistry of *cyclo*‐P_5_^−^ solutions must be revisited, as we could now isolate and fully characterize **C** as its [M(2.2.2‐cryptand)]^+^ (M  = Na, K) salts.

Several approaches to generate the *cyclo*‐P_5_^−^ ion in solution are presently known.^[^ ^4^, ^5^, ^32^, ^33^ ^]^ Generally, *cyclo*‐P_5_^−^ as well as other polyphosphides (P_7_^3−^, P_16_^2−^) form upon activation of P_4_ or P_red_ with alkali metals or strong nucleophiles.^[^ ^34^ ^]^ In the seminal report by Baudler and co‐workers from 1987, *cyclo*‐P_5_^−^ was detected among several other products, which were formed when reacting P_4_ and Na in refluxing diglyme.^[^ ^4^ ^]^ The same group later reported that 0.01 M stock‐solutions of pure [Na(solvent)_n_][*cyclo*‐P_5_] (yield based on P_4_ is around 12%) can be obtained upon activation of P_4_ with Na in THF.^[^ ^5^ ^]^ Most importantly, the precipitation of other sodium polyphosphides is facilitated by adding 18‐crown‐6 and the solution of *cyclo*‐P_5_^−^ in diglyme can be used for follow‐up chemistry. Two decades later, Miluykov and Hey‐Hawkins demonstrated that P_4_ reacts in the presence of dibenzo‐18‐crown‐6 with sodium in refluxing diglyme to give [Na(diglyme)_n_][*cyclo*‐P_5_] in 25% spectroscopic yield, while the second main product of the reaction is Na_3_P_7_, which is insoluble and precipitates from the reaction mixture.^[^ ^32^ ^]^ Nevertheless, all reports so far describe that *cyclo*‐P_5_^−^ is only stable in solution and decomposes upon cooling or when the solvent is removed under vacuum.

With the aim in mind to find a *cyclo*‐P_5_^−^ source, that is thermodynamically and kinetically stable, easily accessible and useable, we investigated a novel synthetic route towards this anionic, aromatic phosphorus cycle using [2.2.2]cryptand for the complexation of the alkali metal cation. In fact, Milyukov et al. suggested a pathway towards *cyclo*‐P_5_^−^, in which higher polyphosphides, for instance Na_3_P_7_ or Na_2_P_16_, are involved. As mentioned above, such species can indeed be detected as byproducts during the synthesis of *cyclo*‐P_5_^−^.^[^ ^32^, ^33^ ^]^ Moreover, Goicoechea and co‐worker reported on the preparation of [K([2.2.2]crypt)]_2_[Co(η^5^‐P_5_){η^2^‐P_2_H(mes)}]_2_.^[^ ^34^ ^]^ This coordination compound is formed upon reaction of K_3_P_7_ with [Co(mes)_2_(PEt_2_Ph)_2_] (mes = 2,4,6‐trimethylphenyl) and contains both a *cyclo‐*P_5_^−^ and a diphosphene ligand. The alkali metal heptaphosphides M_3_P_7_(dme)_x_ (M = Na (**1a**), K(**1b**)) are independently accessible quantitatively from red (P_red_) or white phosphorus (P_4_) and sodium in the presence of naphthalene.^[^ ^35^ ^]^

Motivated by these findings, stoichiometric amounts of **1a** and **1b** were first reacted with [2.2.2]cryptand under reflux in different ethers (THF, diglyme). Much to our surprise, the ^31^P NMR spectra of the yellow‐orange suspensions only revealed a single resonance at *δ* = 468.0 ppm after heating, while the reaction time depends on the boiling point of the solvent. In THF, the highest yield was reached after 72 h, while for diglyme the reaction is already completed after 15 h. This resonance can be attributed to the formation of the *cyclo*‐P_5_^−^ anion as only soluble phosphorus species (Scheme 1a; for the insoluble residue see SI). Interestingly, we noticed that the same reactivity is observed when stoichiometric amounts of white phosphorus, potassium and [2.2.2]cryptand in THF are refluxed for 15 h (Scheme 1b).

**Scheme 1:**
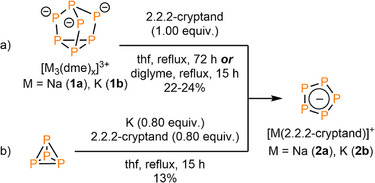
a) Synthesis of [M([2.2.2]cryptand)][*cyclo*‐P_5_] (M = Na (**2a**), K (**2b**)) from M_3_P_7_(dme)_x_ (M = Na (**1a**), K(**1b**)) and (b) from P_4_.

The filtered solutions were subsequently layered with pentane. After storing the samples for 3 days at *T* = –20°C in the freezer, [M([2.2.2]cryptand)][*cyclo*‐P_5_] (M = Na (**2a**), K (**2b**)) crystallized as light‐yellow crystalline blocks, suitable for single crystal X‐ray diffraction. Compound **2a** crystalized from the diglyme solution in the triclinic space group $P\bar{1}$, while **2b** crystalized in the monoclinic space group *P*2_1_/*n*. The molecular structures of **2a** and **2b** in the crystals are depicted in Figures 2 and 3, along with selected bond lengths and angles (For **2a(thf)**: see Figure S2

**Figure 2:**
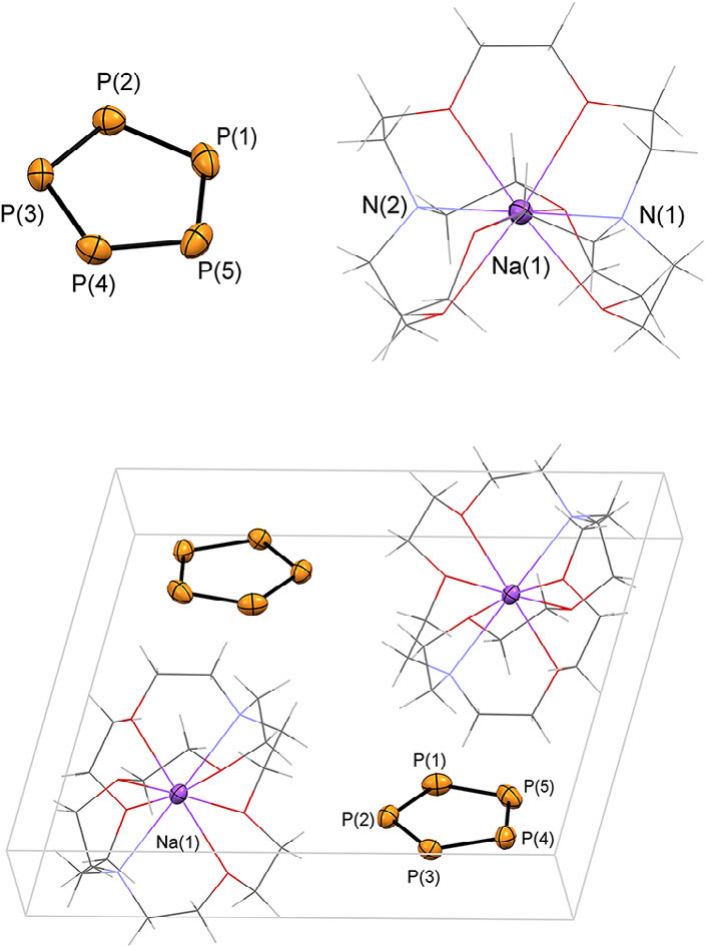
Molecular structure of **2a** in the crystal and the packed unit cell (bottom). Displacement ellipsoids are shown at the 50% probability level. Selected bond lengths (Å) and angles (°): P(1)–P(2) 2.068(1) Å, P(2)–P(3) 2.061(1) Å, P(3)–P(4) 2.082(1) Å, P(4)–P(5) 2.091(1) Å, P(5)–P(1) 2.069(2) Å, N(1)–Na(1) 2.667(3) Å, Na(1)–N(2) 2.703(3) Å, P2–P1–P5 108.27(5)°, P(1)–P(2)–P(3) 108.20(5)°, P(2)–P(3)–P(4) 108.35(5)°, P(3)–P(4)–P(5) 107.31(5)°, P(4)–P(5)–P(1) 107.86(5)°.

**Figure 3:**
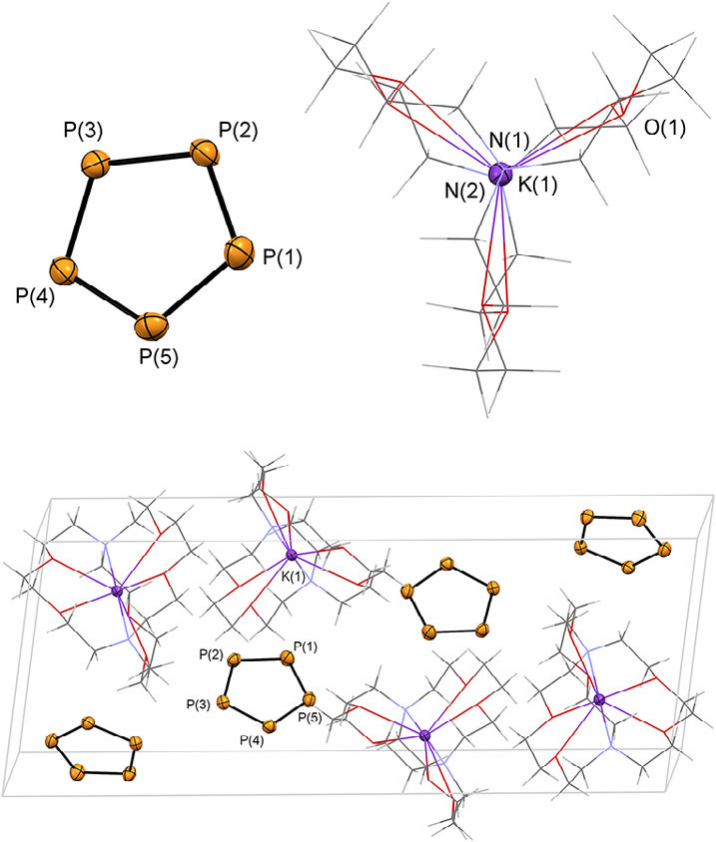
Molecular structure of **2b** in the crystal and the packed unit cell (bottom). Displacement ellipsoids are shown at the 50% probability level. Selected bond lengths (Å) and angle (°): P(1)–P(2) 2.0930(7) Å, P(2)–P(3) 2.0896(7) Å, P(3)–P(4) 2.0837(7) Å, P(4)–P(5) 2.0778(6) Å, P(5)–P(1) 2.0807(7) Å, N(1)–K(1) 3.001(2) Å, K(1)–N(2) 3.0191(1) Å, P2–P1–P5 107.78(3)°, P(1)–P(2)–P(3) 108.25(3)°, P(2)–P(3)–P(4) 107.50(3)°, P(3)–P(4)–P(5) 108.92(3)°, P(4)–P(5)–P(1) 107.55(3)°.

Much to our delight, the crystallographic characterization of the products confirmed the formation of **2a** and **2b** and, for the first time, the stability of the *cyclo*‐P_5_^−^ anion in the solid state. The packed unit cell shows neither significant anion‐cation, nor anion‐anion interactions but only the naked *cyclo*‐P_5_^−^ separated from the weakly coordinating cation. As anticipated spectroscopically and by means of computational chemistry, the isolated *cyclo*‐P_5_^−^ anion reveals a planar structure, which is in accordance with the solid‐state structures of the isolobal cyclopentadiene anion and the valence isoelectronic *cyclo*‐N_5_^−^ anion. The average P─P bond length in the five‐membered phosphorus cycle is 2.074 Å for **2a** and 2.085 Å for **2b**, which is between common P─P‐single (2.21 Å) and P═P‐double (2.02 Å) bonds.^[^ ^36^ ^]^ The average P─P─P angles are 108.00°, as expected for a regular pentagon. Moreover, the P─P bond lengths are in the range of the experimentally determined P─P distances in reported iron and ruthenium *mono*‐P_5_ complexes (2.07–2.12 Å).^[^ ^16^, ^18^ ^]^ The P─P─P─P torsion angles are below 0.05° (**2a**), 1.22°(**2a(thf)**) and 0.26° (**2b**).

The geometry optimization of the *cyclo*‐P_5_^−^ anion performed at the PBE0‐D3/def2‐TZVP level indeed reveals a *D_5_* *_h_* planar structure of the phosphorus cycle with P─P distances of 2.0899 Å and P─P─P angles of 108°, which are perfectly in line with the experimentally observed data. The calculated ^31^P NMR chemical shift of *δ* = 516 ppm for the *cyclo*‐P_5_^−^ anion is also close to the experimental value (see ESI). Figure 4 represents an overview of selected frontier orbitals of the pentaphospholide anion in comparison to the isolobal cyclopentadienide anion. As already reported in literature,^[^ ^17^ ^]^ the *cyclo*‐P_5_^−^ anion acts in the corresponding transition metal π‐complexes as a less good σ‐ and π‐donor, but a much better δ‐acceptor ligand than its Cp^−^ analog (HOMO^−1^, *e*_1_: E = −2.387 eV; LUMO, *e*_2_: E = 2.310 eV).^[^ ^37^ ^]^ Most strikingly, the crystallized salts **2a** and **2b** can be isolated by filtration, followed by vacuum drying. The resulting pale yellow‐orange powders can be stored under argon at room temperature over several weeks.

**Figure 4:**
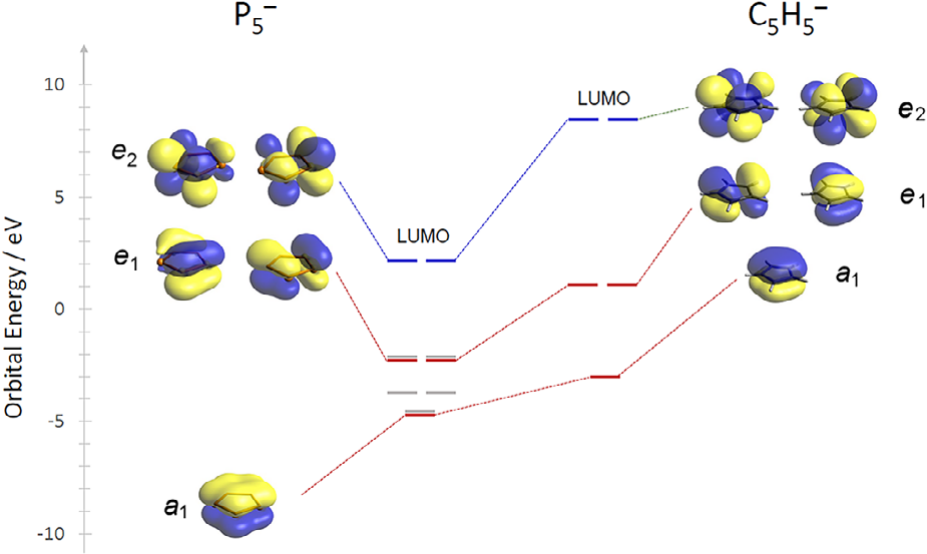
Overview of selected frontier orbitals of the pentaphospholide anion in comparison to the isolobal cyclopentadienide anion.

Moreover, the [M([2.2.2]cryptand)][cyclo‐P_5_] salts can be re‐dissolved in organic solvents, such as THF and CH_2_Cl_2_. While **2a/b** are stable in THF for several weeks, oligomerization/decomposition can be observed after several hours in CH_2_Cl_2_. In this case, an insoluble orange residue is formed, which could not be identified by means of ^31^P NMR spectroscopy. This residue can, however, be filtered off, while the remaining solution contains only **2a/b**. Supported by computational data, the isolation of the [M([2.2.2]cryptand)][*cyclo*‐P_5_] salts allows for the first time additional characterizations of such compounds, both in solution and in the solid state. The UV/Vis spectrum of **2a** in THF shows a broad band between λ = 280 nm and λ = 400 nm (**2b**: λ_max_ = 270 nm), which can be attributed mainly to π→π* transitions in the *cyclo*‐P_5_^−^ anion (Figures 4 and 5 and SI).^[^ ^38^ ^]^ Similar concentration‐dependent spectra were observed by Baudler and co‐worker for NaP_5_/[18]crown‐6/THF solutions.^[^ ^5^ ^]^

**Figure 5:**
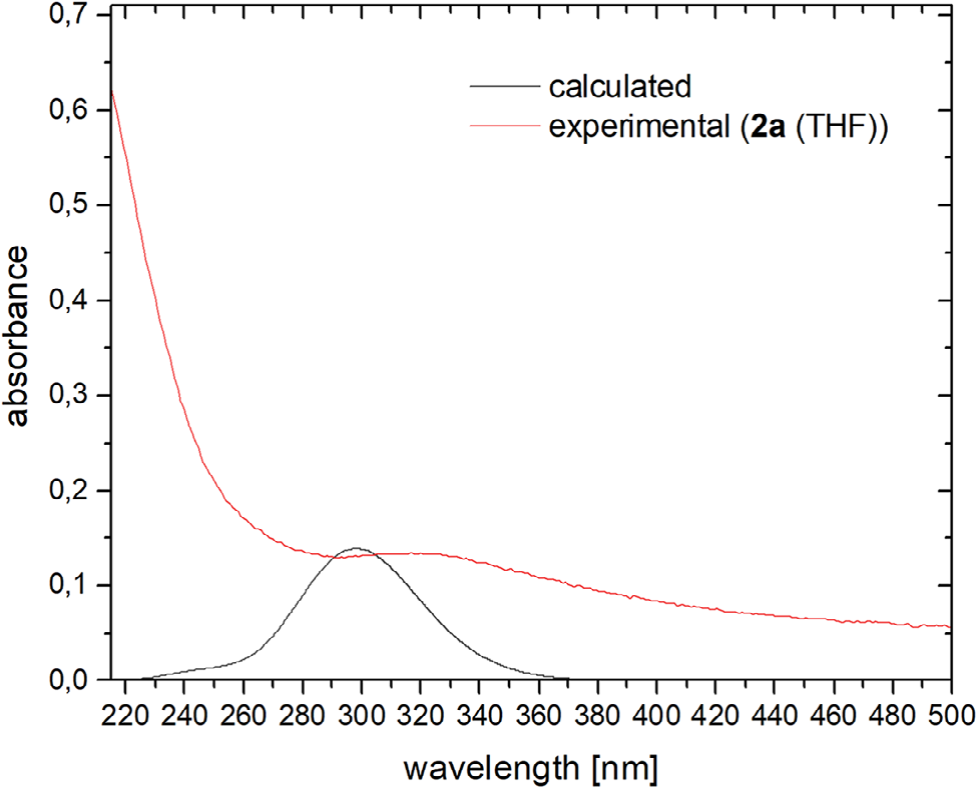
Experimental (red) UV‐Vis spectrum for **2a** recorded in THF solution measured at room temperature and calculated (black) UV‐Vis spectrum.

The powder Raman spectrum of **2a** shows two characteristic bands for the *cyclo*‐P_5_^−^ anion at $\tilde{\nu }=$464 cm^−1^ (Figure 6: ring breathing vibration, calculated value: $\tilde{\nu }=$461 cm^−1^) and at $\tilde{\nu }=$294 cm^−1^ (Figure 6; ring distortion vibration, calculated value: $\tilde{\nu }=$289 cm^−1^).

**Figure 6:**
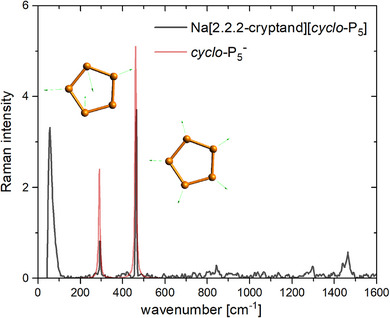
Experimental Raman spectrum of **2a** and calculated Raman spectrum of *cyclo*‐P_5_^−^.

Compound **2a** was also characterized by means of solid‐state ^31^P NMR spectroscopy under magic‐angle spinning (MAS). The spectra, measured at different spin rates (7  and 10 kHz), are depicted in Figure 7.

**Figure 7:**
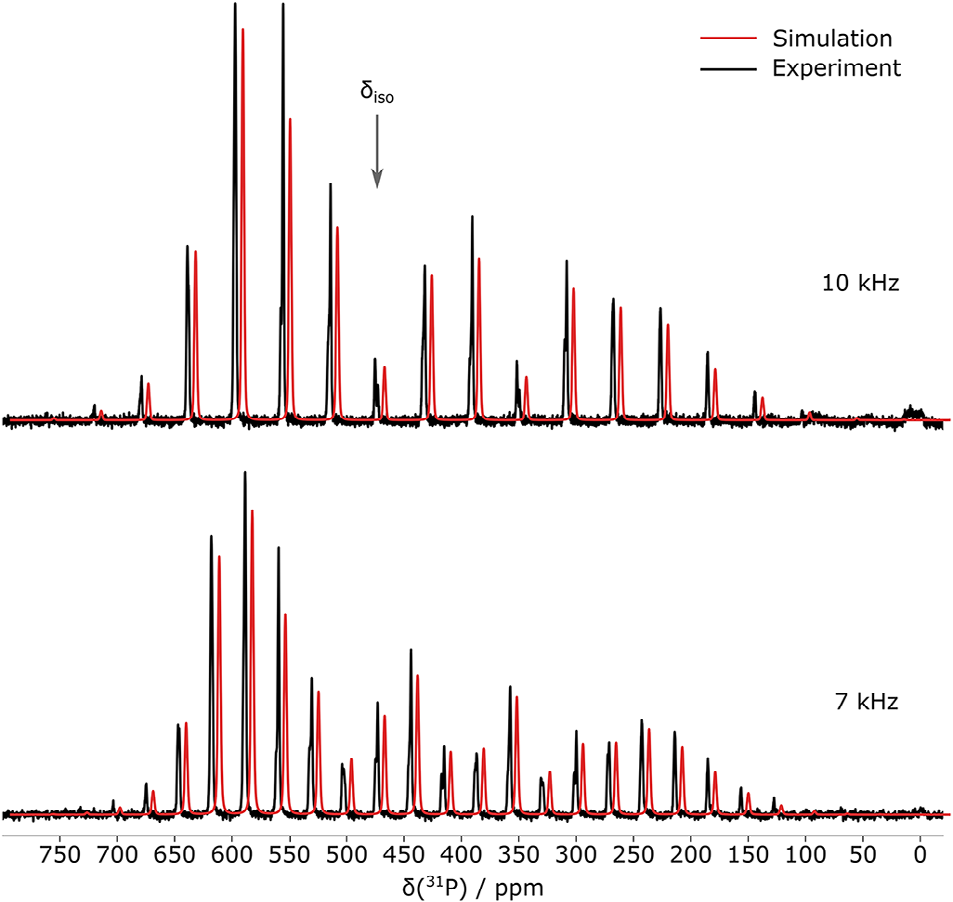
Experimental ^31^P MAS NMR spectra of **2a** at 7 kHz (bottom) and 10 kHz (top) MAS frequency. In addition to the experimental spectra (black), the simulations obtained by a global fit (red) are shown. The simulated spectra are shifted by 1.2 ppm upfield for better visibility. The signal at the isotropic chemical shift of *cyclo*‐P_5_^−^ is marked by an arrow.

Both spectra show a mutual isotropic central band at *δ*_iso_ = 473.3 ppm with a number of rotational sidebands separated by the respective MAS frequency of 7 and 10 kHz, respectively. Spectral simulation revealed a chemical shift anisotropy (CSA) with a reduced anisotropy (*δ_zz_* − *δ*_iso_) of −320.0 ppm and no asymmetry (*η* = 0). The quality of the multi‐frequency fit and the absence of any additional resonances is in line with the observation from crystallographic data that all ^31^P atoms in each *cyclo*‐P_5_^−^ ring as well as both rings in the unit cell are virtually identical, and further displays the high symmetry and purity of the compound. Each signal (i.e., central band and sidebands) features a splitting pattern of ∼2.4 ppm (570 Hz), which lies in the same order of magnitude as ^31^P‐^31^P ^1^ *J*‐couplings reported in literature.^[^ ^39^ ^]^ Note, that in the case of *cyclo*‐P_5_^−^, the expected *J*‐coupling pattern is non‐trivial due to the near‐isochronicity of the five ^31^P nuclei. Perfect isochronicity would render any *J*‐coupling invisible; however, small inequivalences in each nucleus's proximity likely introduce chemical shift deviations of similar magnitude as the observed splitting. This could lead to the observed splitting in an “intermediate‐coupling” situation.^[^ ^40^ ^]^

In view of the rather high stability of **2a/b** both in the solid state and in solution, we examined the preparation of corresponding coordination compounds. For this purpose, we envisaged the synthesis of the known ferrocene derivative [Cp*Fe(*cyclo*‐P_5_)] (**3**) that shows a chemical shift in the ^31^P{^1^H} NMR spectrum at δ = 151.5 ppm. Baudler and co‐worker reported on its synthesis from Li(*cyclo*‐P_5_), LiCp* and FeCl_2_.^[^ ^5^ ^]^ Much to our delight, we found a similar reactivity of **2a/b** compared to the Li(*cyclo*‐P_5_), generated in situ (Scheme 2, and SI).

**Scheme 2:**
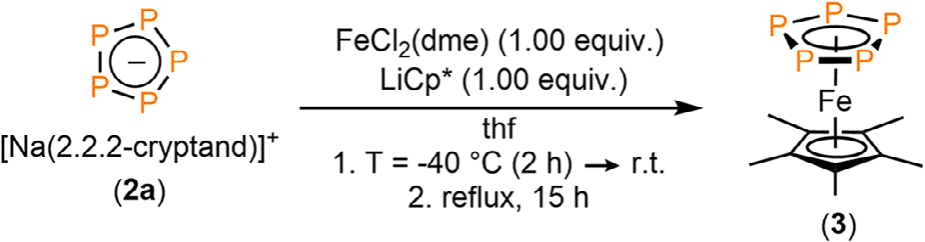
Synthesis of [Cp*Fe(*cyclo*‐P_5_)] (**3**) starting from [Na([2.2.2]cryptand)][*cyclo*‐P_5_], LiCp* and FeCl_2_(dme).

In conclusion, we have developed two new synthetic routes for alkali metal salts of the *cyclo*‐pentaphospholide anion. By using [2.2.2]cryptand, it was possible to generate the *cyclo‐P_5_^−^*‐anion from the alkali metal heptaphosphides M_3_P_7_ (M = Na, K). A similar reactivity was observed when using stoichiometric amounts of white phosphorus, potassium and [2.2.2]cryptand in THF. The isolation as the salts [M(2.2.2‐cryptand)][*cyclo*‐P_5_] (M = Na, K) led for the first time to a crystallographic characterization of both compounds. The fully inorganic cyclopentadienide derivates were additionally investigated by means of theoretical and experimental methods, such as Raman‐, IR‐, UV/Vis‐, as well as solid‐state ^31^P MAS NMR spectroscopy. The stability of the salts [M([2.2.2]cryptand)][*cyclo*‐P_5_] (M = Na, K) allows the handling of the *cyclo*‐P_5_^−^ anion in its uncoordinated form in organic solvents, such as THF and CH_2_Cl_2_. Moreover, the reaction of [Na([2.2.2]cryptand)][*cyclo*‐P_5_] with LiCp* and FeCl_2_ yields the ferrocene derivative [Cp*Fe(*cyclo*‐P_5_)].

## Supporting Information

The authors have cited additional references within the Supporting Information.^[^ ^41^, ^42^, ^43^, ^44^, ^45^, ^46^, ^47^, ^48^, ^49^, ^50^, ^51^, ^52^, ^53^, ^54^, ^55^, ^56^, ^57^, ^58^, ^59^, ^60^, ^61^, ^62^, ^63^, ^64^, ^65^ ^]^ The Supporting Information includes detailed syntheses for all the reported compounds, analytical and spectroscopic data and a summary of the computational techniques used and relevant results.

## Conflict of Interests

The authors declare no conflict of interest.

## Supporting information



Supporting Information

## Data Availability

The data that support the findings of this study are available in the supplementary material of this article.

## References

[1] C. Zhang , C. Sun , B. Hu , C. Yu , M. Lu , Science 2017, 355, 374–376. 28126812 10.1126/science.aah3840

[2] Y. Xu , Q. Wang , C. Shen , Q. Lin , P. Wang , M. Lu , Nature 2017, 549, 78–81. 28847006 10.1038/nature23662

[3] C. Yang , C. Zhang , Z. Zheng , C. Jiang , J. Luo , Y. Du , B. Hu , C. Sun , K. O. Christe , J. Am. Chem. Soc. 2018, 140, 16488–16494. 30392354 10.1021/jacs.8b05106

[4] M. Baudler , D. Düster , D. Ouzounis , Z. Anorg. Allg. Chem. 1987, 544, 87–94.

[5] M. Baudler , S. Akpapoglou , D. Ouzounis , F. Wasgestian , B. Meinigke , H. Budzikiewicz , H. Münster , Angew. Chem. Int. Ed. Engl. 1988, 27, 280–281.

[6] M. Baudler , T. Etzbach , Chem. Ber. 1991, 124, 1159–1160.

[7] T. P. Hamilton , H. F. Schaefer, III , Angew. Chem. Int. Ed. Engl. 1989, 28, 485–486.

[8] R. Janoschek , Chem. Ber. 1989, 122, 2121–2124.

[9] H. Zhai , L. Wang , J. Phys. Chem. A 2002, 106, 5600–5606.

[10] Q. Jin , B. Jin , W. G. Xu , W. Zhu , J. Mol. Struct. THEOCHEM 2005, 713, 113–117.

[11] J. O. C. Jiménez‐Halla , E. Matito , J. Robles , M. Solá , J. Organomet. Chem. 2006, 691, 4359–4366.

[12] R. Gupta , R. K. Bansal , Comput. Theor. Chem. 2016, 1076, 1–10.

[13] I. Alkorta , J. Elguero , Struct. Chem. 2016, 27, 1531–1542.

[14] S. Jain , D. Danovich , S. Radenković , S. Shaik , J. Am. Chem. Soc. 2025, 147, 1092–1100. 39700322 10.1021/jacs.4c14515

[15] O. J. Scherer , J. Schwalb , G. Wolmershäuser , W. Kaim , R. Gross , Angew. Chem. Int. Ed. Engl. 1986, 25, 363–364.

[16] O. J. Scherer , T. Brück , Angew. Chem. 1987, 99, 59.

[17] O. J. Scherer , T. Brück , G. Wolmershäuser , Chem. Ber. 1988, 121, 935–938.

[18] E. Urnèžius , W. W. Brennessel , C. J. Cramer , J. E. Ellis , P. von Ragué Schleyer , Science 2002, 295, 832–834. 11823635 10.1126/science.1067325

[19] B. Rink , O. J. Scherer , G. Wolmershäuser , Chem. Ber. 1995, 128, 71–73.

[20] C. Heinl , E. Peresypkina , G. Balázs , E. Mädl , A. V. Virovets , M. Scheer , Chem. ‐ Eur. J. 2021, 27, 7542–7548. 33543820 10.1002/chem.202100203 PMC8252565

[21] M. Baudler , T. Etzbach , Angew. Chem. Int. Ed. Engl. 1991, 30, 580–582.

[22] C. Schwarzmaier , A. Schindler , C. Heindl , S. Scheuermayer , E. V. Peresypkina , A. V. Virovets , M. Neumeier , R. Gschwind , M. Scheer , Angew. Chem. Int. Ed. Engl. 2013, 52, 10896–10899. 24000137 10.1002/anie.201306146

[23] F. Dielmann , C. Heindl , F. Hastreiter , E. V. Peresypkina , A. V. Virovets , R. M. Gschwind , M. Scheer , Angew. Chem. Int. Ed. Engl. 2014, 53, 13605–13608. 25288480 10.1002/anie.201407120 PMC4501309

[24] M. E. Moussa , B. Attenberger , E. V. Peresypkina , M. Scheer , Dalton Trans. 2018, 47, 1014–1017. 29260175 10.1039/c7dt04286h

[25] C. Riesinger , G. Balázs , M. Bodensteiner , M. Scheer , Angew. Chem. Int. Ed. Engl. 2020, 59, 23879–23884. 32956573 10.1002/anie.202011571 PMC7814675

[26] X. Sun , A. Hinz , S. Schulz , L. Zimmermann , M. Scheer , P. W. Roesky , Chem. Sci. 2023, 14, 4769–4776. 37181779 10.1039/d3sc00806a PMC10171192

[27] S. Reichl , E. Mädl , F. Riedlberger , M. Piesch , G. Balázs , M. Seidl , M. Scheer , Nat. Commun. 2021, 12, 5774. 34599185 10.1038/s41467-021-26002-7 PMC8486752

[28] M. Fleischmann , S. Welsch , H. Krauss , M. Schmidt , M. Bodensteiner , E. V. Peresypkina , M. Sierka , C. Gröger , M. Scheer , Chem. ‐ Eur. J. 2014, 20, 3759–3768. 24615817 10.1002/chem.201304466

[29] H. J. Breunig , N. Burford , R. Rösler , Angew. Chem. Int. Ed. Engl. 2000, 39, 4148–4150. 11093235 10.1002/1521-3773(20001117)39:22<4148::aid-anie4148>3.0.co;2-1

[30] J. Rienmüller , B. Peerless , S. Paul , F. Bruder , W. Wernsdorfer , F. Weigend , S. Dehnen , Nat. Chem. 2025, 10.1038/s41557-024-01713-8. PMC11964920 39833513

[31] A. Petrov , L. Conrad , N. T. Coles , M. Weber , D. Andrae , A. Zagidullin , V. Miluykov , C. Müller , Chem. ‐ Eur. J. 28, e202203056. 36210344 10.1002/chem.202203056 PMC10098531

[32] V. A. Milyukov , A. V. Kataev , O. G. Sinyashin , E. Hey‐Hawkins , Russ. Chem. Bull. Int. Ed. 2006, 55, 1297–1299.

[33] M. Jo , A. Dragulescu‐Andrasi , L. Z. Miller , C. Pak , M. Shatruk , Inorg. Chem. 2020, 59, 5483–5489. 32271557 10.1021/acs.inorgchem.0c00108

[34] C. M. Knapp , B. H. Westcott , M. A. C. Raybould , J. E. McGrady , J. M. Goicoechea , Angew. Chem. Int. Ed. Engl. 2012, 51, 9097–9100. 22847864 10.1002/anie.201203980

[35] M. Cicač‐Hudi , J. Bender , S. H. Schlindwein , M. Bispinghoff , M. Nieger , H. Grützmacher , D. Gudat , Eur. J. Inorg. Chem. 2016, 2016, 649–658.

[36] L. Weber , Chem. Rev. 1992, 92, 1839–1906.

[37] B. M. Gardner , F. Tuna , E. J. L. McInnes , J. McMaster , W. Lewis , A. J. Blake , S. T. Liddle , Angew. Chem. Int. Ed. 2015, 54, 7068–7072. 10.1002/anie.201501728 PMC4517156 25914060

[38] The slight hypsochromic shift and the width of the absorption band for **2b** is slightly shorter compared to **2a**. See for example: D. M. S. Buyens , L. A. Pilcher , E. Roduner , Eds. ChemPhysChem 2021, 22, 2025–2033. 34153151 10.1002/cphc.202100098 PMC8518609

[39] M. Gee , R. E. Wasylishen , P. J. Ragogna , N. Burford , R. McDonald , Can. J. Chem. 2002, 80, 1488–1500.

[40] Other possible reasons for the splitting could be higher order interactions not spun out by MAS (e.g., multi‐spin dipolar effects in the P_5_‐system) or instrumental inefficiencies (e.g., deviation from the magic angle, magnetic field inhomogeneity).

[41] MestReNova 14.1.0‐24037, Mestrelab Reasearch S.L.: 2019.

[42] Bruker , APEX ΙΙΙ. Bruker AXS Inc., Madison, Wisconsin, USA 2019.

[43] Bruker , Apex 4, Bruker AXS Inc. Madison, WI, USA 2021.

[44] G. M, Sheldrick , SADABS, University of Göttingen, Germany 1996.

[45] P., Coppens , Crystallographic Computing, Copenhagen, Muksgaard 1979.

[46] O. V. Dolomanov , L. J. Bourhis , R. J. Gildea , J. a. K. Howard , H. Puschmann , J. Appl. Cryst. 2009, 42, 339–341. 10.1107/S0021889811041161 PMC3236671 22199401

[47] G. M., Sheldrick , Acta Crystallogr. Sect. A: Found. Crystallogr. 2008, A64, 112–122. 10.1107/S0108767307043930 18156677

[48] G. M., Sheldrick , Acta Crystallogr. Sect. C: Struct. Chem. 2015, *C* 71, 3–8. 25567568 10.1107/S2053229614024218 PMC4294323

[49] T. facility , “CheckCIF,” http://checkcif.iucr.org.

[50] *Diamond – Crystal and Molecular Structure Visualization Crystal Impact* – Dr. H. Putz & Dr. K. Brandenburg GbR, Bonn, Germany 2019.

[51] M. J. Frisch , G. W. Trucks , H. B. Schlegel , G. E. Scuseria , M. A. Robb , J. R. Cheeseman , G. Scalmani , V. Barone , G. A. Petersson , H. Nakatsuji , X. Li , M. Caricato , A. V. Marenich , J. Bloino , B. G. Janesko , R. Gomperts , B. Mennucci , H. P. Hratchian , J. V. Ortiz , A. F. Izmaylov , J. L. Sonnenberg , D. Williams‐Young , F. Ding , F. Lipparini , F. Egidi , J. Goings , B. Peng , A. Petrone , T. Henderson , D. Ranasinghe , et al., Gaussian, Inc., Wallingford CT, 2016.

[52] T. Lu , F. Chen , J. Comput. Chem. 2012, 33, 580–592. 22162017 10.1002/jcc.22885

[53] C. Adamo , V. Barone , The J. Chem. Phys. 1999, 110, 6158–6170.

[54] F. Weigend , R. Ahlrichs , Phys. Chem. Chem. Phys. 2005, 7, 3297. 16240044 10.1039/b508541a

[55] S. Grimme , J. Antony , S. Ehrlich , H. Krieg , J. Chem. Phys. 2010, 132, 154104–154104. 20423165 10.1063/1.3382344

[56] S. Grimme , S. Ehrlich , L. Goerigk , J. Comput. Chem. 2011, 32, 1456–1465. 21370243 10.1002/jcc.21759

[57] D. S. Tikhonov , I. Gordiy , D. A. Iakovlev , A. A. Gorislav , M. A. Kalinin , S. A. Nikolenko , K. M. Malaskeevich , K. Yureva , N. A. Matsokin , M. Schnell , ChemPhysChem 2024, 25, e202400547. 39172051 10.1002/cphc.202400547 PMC11614367

[58] F. London , J. Phys. Radium 1937, 8, 397–409.

[59] R. McWeeny , Phys. Rev. 1962, 126, 1028–1034.

[60] R. Ditchfield , Mol. Phys. 1974, 27, 789–807.

[61] K. Wolinski , J. F. Hinton , P. Pulay , J. Am. Chem. Soc. 1990, 112, 8251–8260.

[62] J. R. Cheeseman , G. W. Trucks , T. A. Keith , M. J. Frisch , J. Chem. Phys. 1996, 104, 5497–5509.

[63] C. J. Jameson , A. De Dios , A. K. Jameson , Chem. Phys. Lett. 1990, 167, 575–582.

[64] C. van Wüllen , Phys. Chem. Chem. Phys. 2000, 2, 2137–2144.

[65] S. G. J. van Meerten , W. M. J. Franssen , A. P. M. Kentgens , J. Magn. Reason. 2019, 301, 56–66. 10.1016/j.jmr.2019.02.006 30851666

