# Supplementary material for: Cyclo‐P5 − Revisited: The Surprisingly Stable Uncoordinated Pentaphospholide Anion
Source: Angew Chem Int Ed Engl. 2025 Apr 27;64(24):e202505853. doi: 10.1002/anie.202505853 (PMC12238910; doi:10.1002/anie.202505853)
Supplement: Supplementary file 1 — Supporting Information [file ANIE-64-e202505853-s001.pdf]

Electronic Supplementary Information for the paper entitled:

Cyclo-P_5^- Revisited: The Surprisingly Stable Uncoordinated Pentaphospholide Anion

Moritz J. Ernst, Andrey Petrov, Mirjam Schröder, Björn Corzilius and Christian Müller

E-mail: c.mueller@fu-berlin.de.

Table of contents

Part 1: Synthetic procedures **4**

1. General Information *4*

1.1. Materials *4*

1.2. Physical Measurements *4*

1.3. Precautions *4*

*2. Synthesis of $[P_5][Na([2.2.2]cryptand)]$ (**2a**) from $Na_3P_7(dme)_x$* *5*

*3. Synthesis of $[P_5][K([2.2.2]cryptand)]$ (**2b**) from $K_3P_7(dme)_x$* *6*

*4. Synthesis of $[Cp*Fe(cyclo-P_5)]$ (**3**) from **2a*** *7*

Part 2: Structure elucidation **8**

Table S1. Crystal data and structure determination parameters. 9

Figure S1. Ellipsoid representation (50% probability) of $[P_5][Na([2.2.2]cryptand)]$ (**2a**). 10

Figure S2. Ellipsoid representation (50% probability) of $[P_5][Na([2.2.2]cryptand)]$ (**2a**(thf)). The structure was refined as a twin with two domains which contribute nearly equally with a BASF value of 0.50044. . 10

Figure S3. Ellipsoid representation (50% probability) of $[P_5][K([2.2.2]cryptand)]$ (**2b**). 11

Table S2. Selected bond lengths (Å). 12

Table S3. Selected bond angles (°). 13

Table S3 (continued). Selected bond angles (°). 14

Part 3: Spectral data **15**

Figure S4. ^{31}P NMR spectrum (thf-d_8) of $[P_5][Na([2.2.2]cryptand)]$ (**2a**). 15

Figure S5. $^{31}P\{^1H\}$ NMR spectrum (thf-d_8) of $[P_5][Na([2.2.2]cryptand)]$ (**2a**). 15

Figure S6. ^1H NMR spectrum (thf-d_8) of $[P_5][Na([2.2.2]cryptand)]$ (**2a**). 16

Figure S7. $^{13}C\{^1H\}$ NMR spectrum (thf-d_8) of $[P_5][Na([2.2.2]cryptand)]$ (**2a**). 16

Figure S8. Experimental UV/VIS spectrum of $[P_5][Na([2.2.2]cryptand)]$ (**2a**) recorded in THF solution measured at room temperature. 17

Figure S9. Experimental Raman spectrum of $[P_5][Na([2.2.2]cryptand)]$ (**2a**) recorded at room temperature. 17

Figure S10. Experimental infra-red (IR) spectrum of $[P_5][Na([2.2.2]cryptand)]$ (**2a**) recorded at room temperature. 18

Figure S11. ESI^- MS spectrum of $[P_5][Na([2.2.2]cryptand)]$ (**2a**). 18

Figure S12. ESI^+ MS spectrum of $[P_5][Na([2.2.2]cryptand)]$ (**2a**). 19

Figure S13. ^{31}P solid-state NMR spectrum of $[P_5][Na([2.2.2]cryptand)]$ (**2a**) with a spinning-rate of 10 kHz. 19

Figure S14. Superimposed ^{31}P solid-state NMR spectrum of $[P_5][Na([2.2.2]cryptand)]$ (**2a**) with a spinning-rates of 10 kHz (blue) and 7 kHz (red). 20

Figure S15. Stacked ^{31}P solid-state NMR spectrum of $[P_5][Na([2.2.2]cryptand)]$ (**2a**) with a spinning-rates of 10 kHz (blue) and 7 kHz (red). 20

Figure S16. ^{31}P solid-state NMR (toss cogwheel sideband suppression) spectrum of $[P_5][Na([2.2.2]cryptand)]$ (**2a**) with a spinning-rate of 10 kHz. 21
Figure S17. ^{31}P NMR spectrum (CH_2Cl_2) of $[P_5][K([2.2.2]cryptand)]$ (**2b**). 21
Figure S18. $^{31}P\{^1H\}$ NMR spectrum (CH_2Cl_2) of $[P_5][K([2.2.2]cryptand)]$ (**2b**). 22
Figure S19. ^{1}H NMR spectrum (CH_2Cl_2) of $[P_5][K([2.2.2]cryptand)]$ (**2b**). 22
Figure S20. $^{13}C\{^1H\}$ NMR spectrum (CH_2Cl_2) of $[P_5][K([2.2.2]cryptand)]$ (**2b**). 23
Figure S21. Experimental UV/VIS spectrum of $[P_5][K([2.2.2]cryptand)]$ (**2a**) recorded in THF solution measured at room temperature. 23
Figure S22. $^{31}P\{^1H\}$ NMR spectrum (CH_2Cl_2) of [Cp*Fe(*cyclo*-P_5)]. 24
Figure S23. ^{31}P NMR spectrum (CH_2Cl_2) of [Cp*Fe(*cyclo*-P_5)]. 24
Figure S24. $^{31}P\{^1H\}$ NMR spectrum (thf) of the residue after the filtration of in the synthesis of $[P_5][K([2.2.2]cryptand)]$. 25
Figure S25. $^{31}P\{^1H\}$ NMR spectrum (thf) of the residue after the filtration of in the synthesis of $[P_5][K([2.2.2]cryptand)]$ after the addition of 0.1 mL H_2O. 25
Figure S26. ^{31}P NMR spectrum (thf) of the residue after the filtration of in the synthesis of $[P_5][K([2.2.2]cryptand)]$ after the addition of 0.1 mL H_2O. 26
Figure S27. Experimental Raman spectrum of the residue after the filtration of in the synthesis of $[P_5][K([2.2.2]cryptand)]$ recorded at room temperature. 26
Figure S28. Experimental Raman spectrum of $[P_7][K_3(dme)_{0.12}]$ (**1b**) recorded at room temperature. 27
Figure S29. Experimental Raman spectrum of [2.2.2]cryptand recorded at room temperature. 27
Figure S30. ^{31}P solid-state NMR spectrum of $[P_7][Na_3(dme)_{0.64}]$ (**1a**) with a spinning-rate of 10 kHz. 28

Part 4: Computational and ^{31}P solid-state NMR simulation details 29

1. *General remarks and geometry optimization 29*

Optimized coordinates for *cyclo*-P_5^-: 29
Optimized coordinates for Cp^-: 29

2. *^{31}P NMR shift calculations 30*

3. *^{31}P solid-state NMR simulations 30*

4. *TD-DFT calcuations 30*

Excitation energies and oscillator strengths: 30
Figure S31. Experimental (red) and calculated (black) UV-Vis spectra for $[P_5][M([2.2.2]cryptand)]$ (M = Na (**2a**), K (**2b**)) recorded in thf solution measured at room temperature: 32
Figure S32. Charge density difference plot of for cyclo-P_5^- corresponding to excitation states 7 and 8 (isosurface value: 0.002. red region is electron donating and blue region is electron accepting): 33

References 34

Part 1: Synthetic procedures

1. General Information

1.1. Materials

All commercially available reagents were used as received unless mentioned otherwise. The alkali metal heptaphosphides $M_3P_7(dme)_x$ (M = Na, K) has been prepared by the standard procedure from Grützmacher, Gudat *et al.*[35] Solvents were dried over molecular sieves (3 Å) and degassed by three freeze-pump-thaw cycles. All reactions with air- and moisture-sensitive compounds were performed under an argon atmosphere using standard Schlenk techniques.

1.2. Physical Measurements

The NMR spectra in solution were recorded at 20 °C on the following spectrometers: *JEOL ECS 400* (^{1}H: 399.7 MHz, ^{31}P: 161.8 MHz) and *JEOL JNM-ECA400II* (^{1}H: 400.5 MHz, ^{31}P: 162.1 MHz). The NMR samples were measured at room temperature unless otherwise stated. Chemical shifts (*δ*) are given relative to the signals of the external standards tetramethyl silane (^{1}H, ^{13}C), 85% phosphoric acid (^{31}P). The solid-state NMR spectra were recorded on the following spectrometer: JEOL ECZ600 S (^{31}P: 242.95 MHz). MestReNova 14.1.1 was used to process the NMR spectra.[41] Electrospray ionization mass spectrometry (ESI-MS) was carried out with the ESI-MSD TOF unit of an *Agilent 6210 TOF LC/MS* system. The measurements were performed with a drop of a concentrated dichloromethane solution in methanol. Raman spectra were recorded at room temperature on a *Bruker MultiRAM II* equipped with a low-temperature Ge detector (1064 nm, up to 450 mW, resolution 4 cm^{-1}). IR spectra were recorded on a *Thermo Scientific Nicolet iS10* instrument. Intensities are classified as vs = very strong, s = strong, m = medium, w = weak, vw = very weak, sh = shoulder. UV/Vis spectra were recorded on a *PerkinElmer Lambda 465* photometer with deuterium and tungsten lamps. A quartz cuvette with a Rydberg-Schlenk attachment was used to maintain an argon atmosphere. The UV/Vis spectra were recorded in a THF solution with a 2 x 10^{-4} Molar solution.

1.3. Precautions

White phosphorus is toxic and extremely pyrophoric. Special safety precautions must be considered: work in pairs, available and ready to use fire extinguisher and sand. Traces of white phosphorus can be quenched by an aqueous $CuSO_4$ solution.

2. Synthesis of $[P_5][Na([2.2.2]cryptand)]$ (2a) from $Na_3P_7(dme)_x$

500 mg (1.45 mmol, 1.00 equiv.) of $Na_3P_7(dme)_{0.64}$ and 544 mg (1.45 mmol, 1.00 equiv.) of [2.2.2]cryptand were suspended in 15 mL diglyme. The reaction mixture was refluxed for 15 h. The suspension was filtered over microfiber glass filter and the residue was extracted with thf (5 mL). The organic phases were combined and layered with Et_2O (5 mL) and pentane (50 mL). Storing at −20 °C for 4 days yielding $[P_5][Na([2.2.2]cryptand)]$ as pale-yellow crystals suitable for X-ray analysis. Filtration and drying in high vacuum gives **2a** as an orange-yellow powder with a yield of 24% (192 mg, 346 µmol).

Compound **2a** is stable as a powder under inert atmosphere at room temperature. It can be redissolved in organic solvents (tetrahydrofuran, dichloromethane, acetonitrile and others). Nevertheless, depending on the solvent after multiple hours or some days in solution oligomerization/decomposition is observed, yielding an insoluble orange residue which can't be detected by the ^{31}P NMR but can be filtered off, yielding the pure compound **2a** in solution.

^{31}P NMR (thf-d_8, rt): 468.0 ppm (s).[Fig. S4]

^{31}P{^{1}H} NMR (thf-d_8, rt): 468.0 ppm (s).[Fig. S5]

^{1}H NMR (thf-d_8, rt): 3.58 (s, 12H, $N(CH_2CH_2OC\underline{\mathit{H}}_2C\underline{\mathit{H}}_2OCH_2CH_2)_3N$), 3.54 (t, $^{3}J_{HH}$ = 4.85 Hz, 12H, $N(CH_2C\underline{\mathit{H}}_2OCH_2CH_2OC\underline{\mathit{H}}_2CH_2)_3N$), 2.61 ppm (t, $^{3}J_{HH}$ = 4.85 Hz, 12H, $N(C\underline{\mathit{H}}_2CH_2OCH_2CH_2OCH_2C\underline{\mathit{H}}_2)_3N$).[Fig. S6]

^{13}C{^{1}H} NMR (thf-d_8, rt): 69.44 (s, 6C, $N(CH_2CH_2O\underline{\mathit{C}}H_2\underline{\mathit{C}}H_2OCH_2CH_2)_3N$), 68.62 (s, 6C, $N(CH_2\underline{\mathit{C}}H_2OCH_2CH_2O\underline{\mathit{C}}H_2CH_2)_3N$), 53.89 ppm (s, 6C, $N(\underline{\mathit{C}}H_2CH_2OCH_2CH_2OCH_2\underline{\mathit{C}}H_2)_3N$).[Fig. S7]

Raman (rt): ṽ =294.43(m), 464.47 (vs), 842.56 (vw), 1296.76 (vw), 1463.53 (w), 2821.80 (w), 2877.08 (m), 2908.02 (sh), 2949.73 cm^{-1} (m).[Fig. S9]

IR (rt): ṽ = 742,38 (w), 817,98 (s), 925,09 (s), 1097,47 (vs), 1262,62 (m), 1299,71 (s), 1355,82 (s), 1454,03 (s), 2814,78 (m), 2863,76 (m), 2907,00 (sh), 2963,62 cm^{-1} (m).[Fig. S10]

ESI^--TOF: m/z = 154.8717 (P_5^-, calculated: 154.8688).[Fig. S11]

ESI$^+$-TOF: m/z = 377.2650 ([$C_{18}N_2H_{36}O_6$]H^+, calculated: 376.2652, 16.23%), 399.2468 ([$C_{18}N_2H_{36}O_6$]H^+, calculated: 399.2471, 100.00%), 415.2203([$C_{18}N_2H_{36}O_6$]H^+, calculated: 415.2211, 13.29%).$^{\text{Fig. S12}}$

^{31}P NMR (solid-state, rt): 475.2 (s), 472.7 ppm (sh).$^{\text{Fig. S13}}$

3. Synthesis of [P_5][K([2.2.2]cryptand)] (2b) from $K_3P_7(dme)_x$

Method A: 30.0 mg (76.1 µmol, 1.00 equiv.) of $K_3P_7(dme)_{0.12}$ and 28.7 mg (76.1 mmol, 1.0 equiv.) of [2.2.2]cryptand were combined in 8 mL thf. The reaction mixture was refluxed for 72 h. Afterwards, the suspension was filtered and the residue was extracted with thf (2 mL). The organic phases were combined and layered with Et_2O (5 mL) and pentane (25 mL). Storing at −20 °C for 4 days yielding [P_5][K([2.2.2]cryptand)] as pale-yellow crystals suitable for X-ray analysis. Filtration and drying in high vacuum gives **2b** as an orange-yellow powder with a yield of 22% (9.55 mg, 16.7 µmol).

The yield of 22% can be explained by the occurrence of different insoluble polyphosphides. During the reaction and after the work-up no soluble by-products were observed spectroscopically (^{31}P NMR, ESI-MS). During the reaction formally a $\{P_2\}^{2-}$ fragment is lost. This probably reacts with other heptaphosphide ions to higher polyphosphides which precipitated during the reaction. The precipitated is insoluble in common organic solvents, reactive towards alcohols and water and was analysed by Raman spectroscopy (Figure S27). The observed bands could not be assigned to reported anionic polyphosphorus compounds or to red phosphorus. Upon addition of water to the residue, multiple phosphorus species, mainly polyphosphanes, are formed, as evident from the corresponding ^{31}P and ^{31}P{^{1}H} NMR spectra (Figures S24−S26). The signals occur in the range of δ(ppm) = +50 to −250 and depict high order ^{31}P-^{31}P coupling patterns.

Method B: 69.0 mg (557 µmol, 1.25 equiv.) of white phosphorus (P_4), 17.4 mg (446 µmol, 1.00 equiv.) of potassium and 168 mg (446 µmol, 1.00 equiv.) [2.2.2]cryptand were suspended in 8 mL thf. The reaction mixture was refluxed for 15 h. The suspension was filtered and the residue was extracted with thf (5 mL). The organic phases were combined and layered with pentane (30 mL). Storing at −20 °C for 4 days, followed by filtration and drying in high vacuum gives **2b** as an orange-yellow powder with a yield of 13% (32.5 mg, 57.0 µmol).

The stability and the spectroscopic properties of **2b** are similar to the properties of compound **2a** (see above in 2.).

^{31}P NMR (CD_2Cl_2, rt): 467.1 ppm (s).[Fig. S17]

^{31}P{^{1}H} NMR (CD_2Cl_2, rt): 467.0 ppm (s).[Fig. S18]

^{1}H NMR (CD_2Cl_2, rt): 3.58 (s, 12H, $N(CH_2CH_2OC\underline{\mathit{H}}_2C\underline{\mathit{H}}_2OCH_2CH_2)_3N$), 3.51 (t, $^3J_{HH}$ = 4.66 Hz, 12H, $N(CH_2C\underline{\mathit{H}}_2OCH_2CH_2OC\underline{\mathit{H}}_2CH_2)_3N$), 2.62 ppm (t, $^3J_{HH}$ = 4.66 Hz, 12H, $N(C\underline{\mathit{H}}_2CH_2OCH_2CH_2OCH_2C\underline{\mathit{H}}_2)_3N$).[Fig. S19]

^{13}C{^{1}H} NMR (CD_2Cl_2, rt): 70.78 (s, 6C, $N(CH_2CH_2O\underline{\mathit{C}}H_2\underline{\mathit{C}}H_2OCH_2CH_2)_3N$), 67.81 (s, 6C, $N(CH_2\underline{\mathit{C}}H_2OCH_2CH_2O\underline{\mathit{C}}H_2CH_2)_3N$), 54.08 ppm (s, 6C, $N(\underline{\mathit{C}}H_2CH_2OCH_2CH_2OCH_2$-$\underline{\mathit{C}}H_2)_3N$).[Fig. S20]

4. Synthesis of [Cp*Fe(*cyclo*-P_5)] (3) from 2a

[Cp*Fe(*cyclo*-P_5)] was synthesised by a slightly modified reported procedure.[5] A pre-cooled (T = −40 °C) suspension of Cp*Li (10.3 mg, 72.2 µmol, 1.00 equiv.) in 4 mL of thf was slowly added to the cold (T = −40 °C) suspension of $FeCl_2$(dme) (15.7 mg, 72.2 µmol, 1.00 equiv.) in 4 mL of thf. After the addition was complete, the reaction mixture was stirred at T = −40 °C for two hours. Afterwards, the cold (T = −40 °C) thf solution of 40.0 mg (72.2 µmol, 1.00 equiv.) [P_5][Na([2.2.2]cryptand)] (2a) was added dropwise to the reaction mixture. After the addition was complete, the reaction was allowed to slowly warm up to reflux and stirred at refluxed for 15 h. The resulting suspension was filtered and the solvent was removed under reduced pressure. The crude black solid was sublimed (T = 18 °C, 10^{-3} mbar) to give an orange powder of **3** with a yield of 52% (13.0 mg, 37.6 µmol). The spectroscopic analysis agrees with the reports of Baudler and Scherer.[5,15]

^{31}P NMR (CH_2Cl_2, rt): 151.5 ppm (s).[Fig. S22]

^{31}P{^{1}H} NMR (CH_2Cl_2, rt): 151.5 ppm (s).[Fig. S23]

Part 2: Structure elucidation

Single crystal x-ray diffraction data was collected on a *Bruker D8 Venture* fitted with a Photon II CMOS Detector with Mo K_α radiation (λ = 0.71073 Å) from an *IμS* micro-source, performing φ-and ω-scans. Data collection and processing was handled using the *Bruker APEX3* and *Bruker APEX4* software packages.[42,43] Absorption corrections were carried out by the multiscan method.[44,45] Structures were solved and refined in Olex2[46] with the SHELX program package.[47,48] All non-hydrogen atoms were refined anisotropically, all hydrogen atoms were included into the model at geometrically calculated positions and refined using a riding model. Structures were checked with checkCIF.[49] Selected crystallographic can be found in Table S2 below. The representation of molecular structures was done using the program DIAMOND 4.2.2.[50] Some remaining crystallographic problems are commented on in the respective *.cif* file and in captions to the illustrations of the structures in Figure S1-S3. The CCDC entries 2423302 (**2a**), 2423300 (**2a**(thf)), and 2423301 (**2b**) contain the supplementary crystallographic data for this article. This data can be obtained free of charge from The Cambridge Crystallographic Data Centre via www.ccdc.cam.ac.uk/structures.

Table S1. Crystal data and structure determination parameters.

	$[P_5][Na([2.2.2]cryptand)]$	$[P_5][Na([2.2.2]cryptand)](thf)$	$[P_5][K([2.2.2]cryptand)]$
Compound number	**2a**	**2a**(thf)	**2b**
Empirical formula	$C_{18}H_{36}N_2NaO_6P_5$	$C_{22}H_{44}N_2NaO_7P_5$	$C_{18}H_{36}KN_2O_6P_5$
Formula weight	554.33	626.43	570.44
Temperature/K	100.00	100.00	100.00
Crystal system	triclinic	monoclinic	monoclinic
Space group	P−1	$P2_1$	$P2_1/n$
a/Å	8.8328(3)	8.7047(3)	10.0980(4)
b/Å	11.2765(4)	19.2505(7)	10.9340(4)
c/Å	14.6326(5)	10.1325(4)	25.8531(10)
α/°	74.9010(10)	90	90
β/°	85.1420(10)	113.8560(10)	98.0260(10)
γ/°	80.3370(10)	90	90
Volume/Å^3	1385.89(8)	1552.84(10)	2826.52(19)
Z	2	2	4
ρ_{calc}/g cm^{-3}	1.328	1.340	1.340
μ/mm^{-1}	0.379	0.349	0.504
F(000)	584.0	664.0	1200.0
Crystal size/mm^3	0.109 × 0.076 × 0.032	0.243 × 0.16 × 0.102	0.177 × 0.128 × 0.086
Radiation	MoKα (λ = 0.71073)	MoKα (λ = 0.71073)	MoKα (λ = 0.71073)
Θ range for data collection/°	3.784 to 53.512	4.232 to 52.838	4.05 to 56.61
Index ranges	−11 ≤ h ≤ 11, −14 ≤ k ≤ 14, −18 ≤ l ≤ 18	−10 ≤ h ≤ 9, 0 ≤ k ≤ 24, 0 ≤ l ≤ 12	−11 ≤ h ≤ 13, −14 ≤ k ≤ 14, −34 ≤ l ≤ 34
Reflections collected	54048	3274	95129
Independent reflections	5894 [R_{int} = 0.0559, R_{sigma} = 0.0267]	3274 [R_{int} = ?, R_{sigma} = 0.0178]	7032 [R_{int} = 0.0364, R_{sigma} = 0.0163]
Data/restraints/parameters	5894/0/289	3274/1/335	7032/0/289
Goodness-of-fit on F^2	1.107	1.106	1.086
Final R indexes [I>=2σ (I)]	R_1 = 0.0501, wR_2 = 0.1175	R_1 = 0.0293, wR_2 = 0.0735	R_1 = 0.0328, wR_2 = 0.0746
Final R indexes [all data]	R_1 = 0.0753, wR_2 = 0.1437	R_1 = 0.0319, wR_2 = 0.0767	R_1 = 0.0418, wR_2 = 0.0829
Largest diff. peak/hole / e Å^{-3}	0.50/−0.54	0.36/−0.27	0.40/−0.35
Diffractometer	Bruker D8 Venture	Bruker D8 Venture	Bruker D8 Venture
CCDC access code	2423302	2423300	2423301

Figure S1. Ellipsoid representation (50% probability) of [P_5][Na([2.2.2]cryptand)] (**2a**).

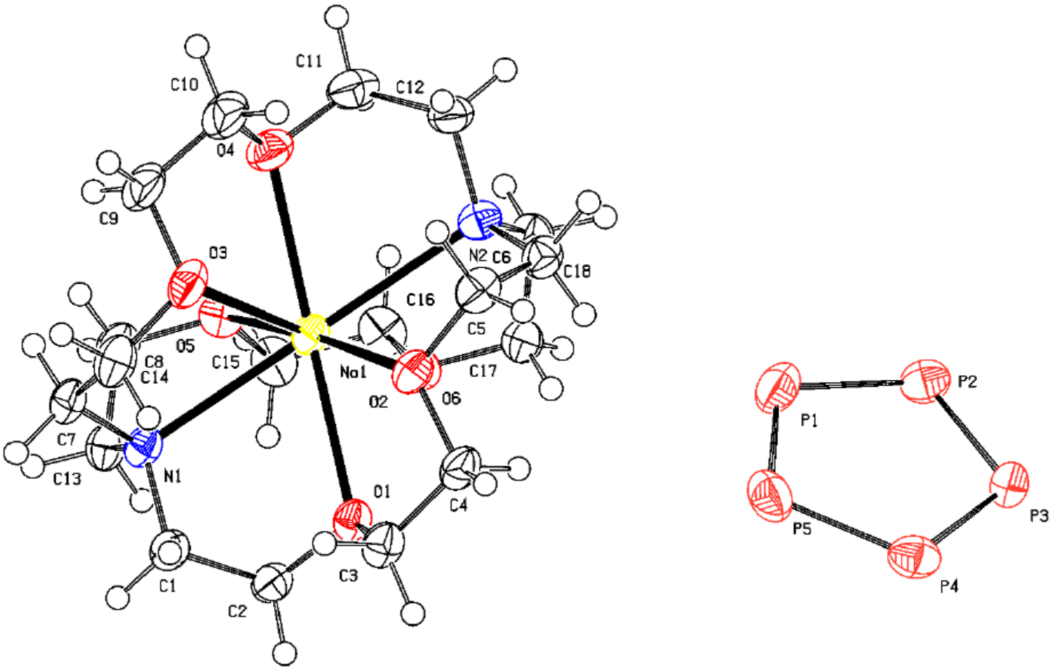

Figure S2. Ellipsoid representation (50% probability) of [P_5][Na([2.2.2]cryptand)] (**2a**(thf)). The structure was refined as a twin with two domains which contribute nearly equally with a BASF value of 0.50044.

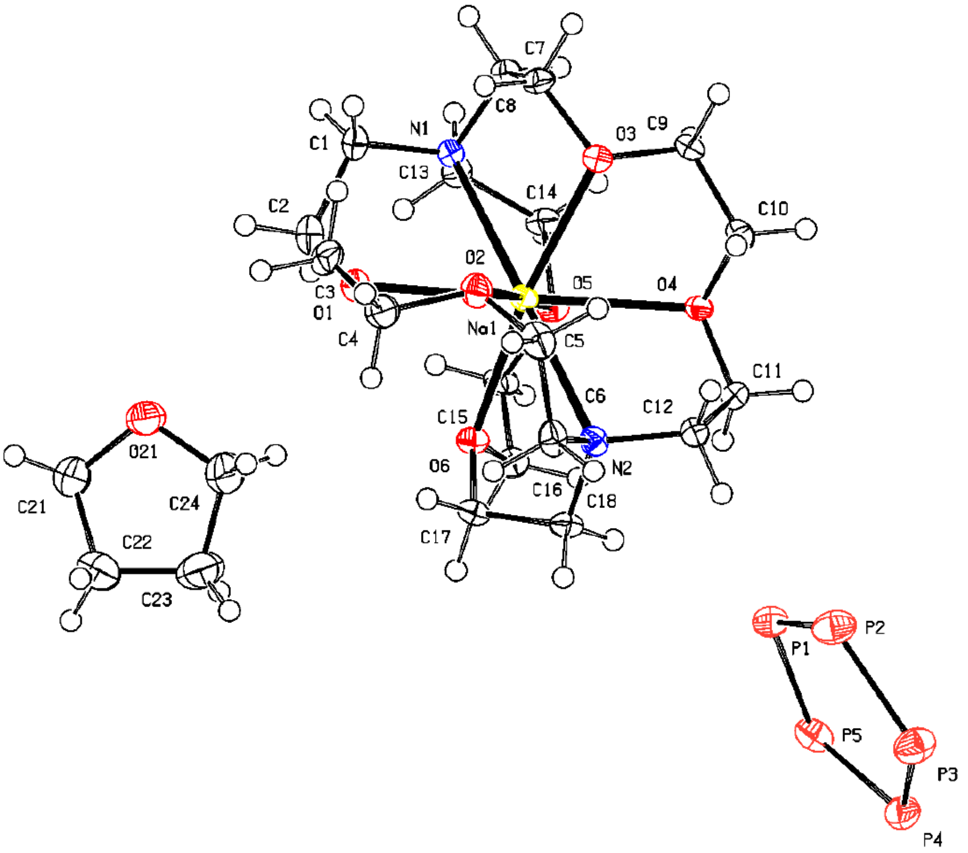

Figure S3. Ellipsoid representation (50% probability) of [P_5][K([2.2.2]cryptand)] (**2b**).

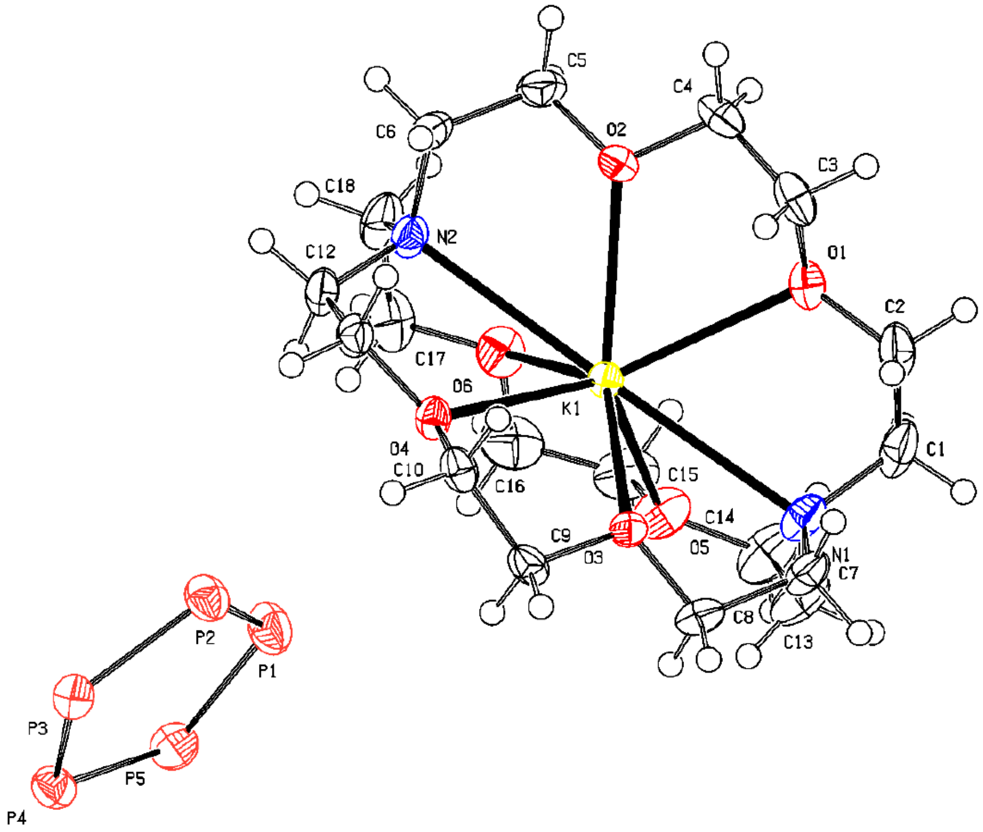

Table S2. Selected bond lengths (Å).

Bond			$[P_5][Na([2.2.2]cryptand)]$ (**2a**) [Å]	$[P_5][Na([2.2.2]cryptand)](thf)$ (**2a**(thf) [Å]	$[P_5][K([2.2.2]cryptand)]$ (**2b**) [Å]
Na1/K1	–	O1	2.540(2)	2.488(3)	2.8222(12)
Na1/K1	–	O2	2.510(2)	2.504(3)	2.7811(12)
Na1/K1	–	O3	2.549(2)	2.391(3)	2.8233(11)
Na1/K1	–	O4	2.495(2)	2.559(3)	2.7780(11)
Na1/K1	–	O5	2.540(2)	2.527(3)	2.8384(13)
Na1/K1	–	O6	2.480(2)	2.389(3)	2.8337(12)
Na1/K1	–	N1	2.667(3)	2.732(4)	3.0010(15)
Na1/K1	–	N2	2.703(3)	2.727(4)	3.0191(14)
O1	–	C2	1.431(3)	1.430(5)	1.425(2)
O1	–	C3	1.423(3)	1.423(5)	1.421(2)
O2	–	C4	1.420(3)	1.427(5)	1.419(2)
O2	–	C5	1.421(4)	1.430(5)	1.420(2)
O3	–	C8	1.424(4)	1.440(5)	1.4246(19)
O3	–	C9	1.431(4)	1.427(5)	1.4209(19)
O4	–	C10	1.422(4)	1.415(5)	1.4196(18)
O4	–	C11	1.431(4)	1.429(5)	1.4279(19)
O5	–	C14	1.423(4)	1.441(5)	1.418(3)
O5	–	C15	1.425(4)	1.439(5)	1.424(2)
O6	–	C16	1.429(3)	1.430(4)	1.432(2)
O6	–	C17	1.428(3)	1.423(5)	1.419(2)
N1	–	C1	1.473(3)	1.468(5)	1.468(3)
N1	–	C7	1.469(3)	1.476(5)	1.476(2)
N1	–	C13	1.471(3)	1.459(5)	1.473(2)
N2	–	C6	1.474(4)	1.471(5)	1.473(2)
N2	–	C12	1.466(4)	1.473(5)	1.468(2)
N2	–	C18	1.473(3)	1.478(5)	1.472(2)
C1	–	C2	1.514(4)	1.515(6)	1.497(3)
C3	–	C4	1.501(4)	1.497(5)	1.492(3)
C5	–	C6	1.507(4)	1.519(6)	1.505(3)
C7	–	C8	1.514(4)	1.514(6)	1.504(3)
C9	–	C10	1.484(5)	1.509(6)	1.495(2)
C11	–	C12	1.513(4)	1.520(5)	1.511(2)
C13	–	C14	1.521(4)	1.506(5)	1.505(3)
C15	–	C16	1.497(4)	1.488(6)	1.482(3)
C17	–	C18	1.521(4)	1.519(5)	1.499(3)
P1	–	P2	2.0684(13)	2.0865(17)	2.0930(7)
P1	–	P5	2.0688(15)	2.0932(14)	2.0896(7)
P2	–	P3	2.0608(12)	2.0863(17)	2.0837(7)
P3	–	P4	2.0815(12)	2.0858(17)	2.0778(6)
P4	–	P5	2.0907(14)	2.0884(16)	2.0807(7)
O21	–	C21		1.416(5)	
O21	–	C24		1.429(5)	
C21	–	C22		1.520(7)	
C22	–	C23		1.486(8)	
C23	–	C24		1.483(7)	

Table S3. Selected bond angles (°).

Angle	[P_5][Na([2.2.2]cryptand)] (**2a**) [°]	[P_5][Na([2.2.2]cryptand)](thf) (**2a**(thf) [°]	[P_5][K([2.2.2]cryptand)] (**2b**) [°]
O1 – Na1/K1 – O3	107.58(8)	65.93(9)	92.64(3)
O1 – Na1/K1 – O5	107.67(7)	175.09(12)	99.82(4)
O1 – Na1/K1 – N1	69.27(7)	107.73(11)	121.57(4)
O1 – Na1/K1 – N2	110.70(7)	68.12(10)	60.12(4)
O2 – Na1/K1 – O1	65.78(7)	110.74(11)	118.68(4)
O2 – Na1/K1 – O3	78.59(7)	109.24(11)	59.93(4)
O2 – Na1/K1 – O5	171.99(8)	173.55(12)	116.30(4)
O2 – Na1/K1 – N1	111.24(8)	110.05(11)	138.15(4)
O2 – Na1/K1 – N2	68.46(7)	68.42(11)	97.49(4)
O3 – Na1/K1 – N1	68.75(7)	109.96(11)	119.63(4)
O3 – Na1/K1 – N2	110.97(8)	79.58(11)	59.25(4)
O4 – Na1/K1 – O1	172.34(8)	67.47(10)	99.65(4)
O4 – Na1/K1 – O2	107.54(8)	104.43(12)	141.43(4)
O4 – Na1/K1 – O3	66.49(8)	70.14(11)	60.52(4)
O4 – Na1/K1 – O5	79.29(7)	109.57(12)	120.44(4)
O4 – Na1/K1 – N1	111.41(8)	113.91(11)	129.96(3)
O4 – Na1/K1 – N2	68.57(7)	67.14(10)	93.14(3)
O5 – Na1/K1 – O3	108.42(7)	77.11(8)	60.51(3)
O5 – Na1/K1 – N1	68.96(7)	67.31(10)	124.32(4)
O5 – Na1/K1 – N2	111.36(8)	114.19(11)	101.82(4)
O6 – Na1/K1 – O1	78.14(7)	79.01(11)	120.45(4)
O6 – Na1/K1 – O2	107.26(7)	108.89(12)	60.64(3)
O6 – Na1/K1 – O3	173.32(8)	169.94(10)	59.98(4)
O6 – Na1/K1 – O4	108.11(8)	103.92(11)	120.23(4)
O6 – Na1/K1 – O5	65.98(7)	67.66(10)	60.22(4)
O6 – Na1/K1 – N1	111.09(8)	110.60(11)	119.06(4)
O6 – Na1/K1 – N2	69.22(7)	69.97(11)	60.88(4)
N1 – Na1/K1 – N2	179.65(8)	178.45(11)	178.68(4)
C2 – O1 – Na1/K1	110.70(15)	113.3(2)	119.79(10)
C3 – O1 – Na1/K1	113.50(17)	115.8(2)	109.93(9)
C3 – O1 – C2	113.2(2)	112.5(3)	111.45(14)
C4 – O2 – Na1/K1	115.05(16)	114.4(2)	118.80(10)
C4 – O2 – C5	114.1(2)	113.3(3)	112.33(13)
C5 – O2 – Na1/K1	111.58(16)	112.2(2)	120.72(10)
C8 – O3 – Na1	111.54(17)	111.1(2)	116.36(9)
C8 – O3 – C9	113.5(2)	115.2(2)	113.30(9)
C9 – O3 – Na1/K1	111.83(19)	115.0(3)	111.67(12)
C10 – O4 – Na1/K1	114.4(2)	110.6(2)	115.49(9)
C10 – O4 – C11	113.5(2)	113.2(3)	111.06(12)
C11 – O4 – Na1/K1	112.32(17)	113.9(2)	119.26(9)
C14 – O5 – Na1/K1	111.40(16)	114.3(2)	118.71(12)
C14 – O5 – C15	113.5(2)	111.1(2)	111.02(16)
C15 – O5 – Na1/K1	113.53(17)	113.6(3)	114.44(11)
C16 – O6 – Na1/K1	115.24(17)	114.8(2)	113.57(11)
C17 – O6 – Na1/K1	111.52(15)	111.9(2)	114.99(10)

Table S3 (continued). Selected bond angles (°).

Angle	$[P_5][Na([2.2.2]cryptand)]$ (**2a**) [°]	$[P_5][Na([2.2.2]cryptand)](thf)$ (**2a**(thf) [°]	$[P_5][K([2.2.2]cryptand)]$ (**2b**) [°]
C17 − O6 − C16	113.5(2)	115.0(3)	110.17(15)
C1 − N1 − Na1/K1	108.12(16)	107.6(2)	108.79(10)
C7 − N1 − Na1/K1	108.48(17)	111.0(3)	109.65(15)
C7 − N1 − C1	111.0(2)	105.5(2)	110.32(16)
C7 − N1 − C13	110.3(2)	109.0(2)	109.45(10)
C13 − N1 − Na1/K1	108.60(16)	111.2(3)	109.64(12)
C13 − N1 − C1	110.3(2)	112.3(3)	108.96(15)
C6 − N2 − Na1/K1	108.04(17)	107.7(2)	111.09(10)
C12 − N2 − Na1/K1	108.46(17)	110.4(3)	107.37(9)
C12 − N2 − C6	111.0(2)	111.1(3)	109.79(14)
C12 − N2 − C18	111.0(2)	109.8(2)	110.22(14)
C18 − N2 − Na1/K1	107.22(16)	111.8(3)	108.45(10)
C18 − N2 − C6	110.9(2)	105.8(2)	109.88(14)
N1 − C1 − C2	112.1(2)	111.8(3)	113.69(15)
O1 − C2 − C1	112.8(2)	111.9(3)	110.02(15)
O1 − C3 − C4	107.8(2)	107.6(3)	109.40(14)
O2 − C4 − C3	107.6(2)	107.6(3)	108.85(14)
O2 − C5 − C6	112.7(2)	112.1(3)	108.76(13)
N2 − C6 − C5	111.5(2)	112.1(3)	113.51(14)
N1 − C7 − C8	112.1(2)	111.3(3)	113.21(15)
O3 − C8 − C7	112.5(2)	112.8(3)	109.00(13)
O3 − C9 − C10	108.5(3)	106.6(3)	109.19(13)
O4 − C10 − C9	108.1(3)	108.0(3)	108.70(13)
O4 − C11 − C12	112.9(3)	112.9(3)	108.50(13)
N2 − C12 − C11	111.9(2)	111.4(3)	113.37(13)
N1 − C13 − C14	112.0(2)	112.9(3)	113.46(17)
O5 − C14 − C13	112.6(2)	112.1(3)	109.26(16)
O5 − C15 − C16	107.6(2)	107.0(3)	109.38(17)
O6 − C16 − C15	107.5(2)	107.4(3)	109.82(16)
O6 − C17 − C18	112.3(2)	113.4(3)	109.66(16)
N2 − C18 − C17	111.4(2)	110.9(3)	114.41(15)
P2 − P1 − P5	108.27(5)	108.14(7)	107.78(3)
P3 − P2 − P1	108.20(5)	107.69(7)	108.25(3)
P2 − P3 − P4	108.35(5)	108.46(6)	107.50(3)
P3 − P4 − P5	107.31(5)	107.79(6)	108.92(3)
P1 − P5 − P4	107.86(5)	107.90(7)	107.55(3)
C21 − O21 − C24		108.6(3)	
O21 − C21 − C22		105.3(3)	
C23 − C22 − C21		102.6(4)	
C24 − C23 − C22		103.4(4)	
O21 − C24 − C23		108.3(4)	

Part 3: Spectral data

Figure S4. ^{31}P NMR spectrum (thf-d_8) of $[P_5][Na([2.2.2]cryptand)]$ (**2a**).

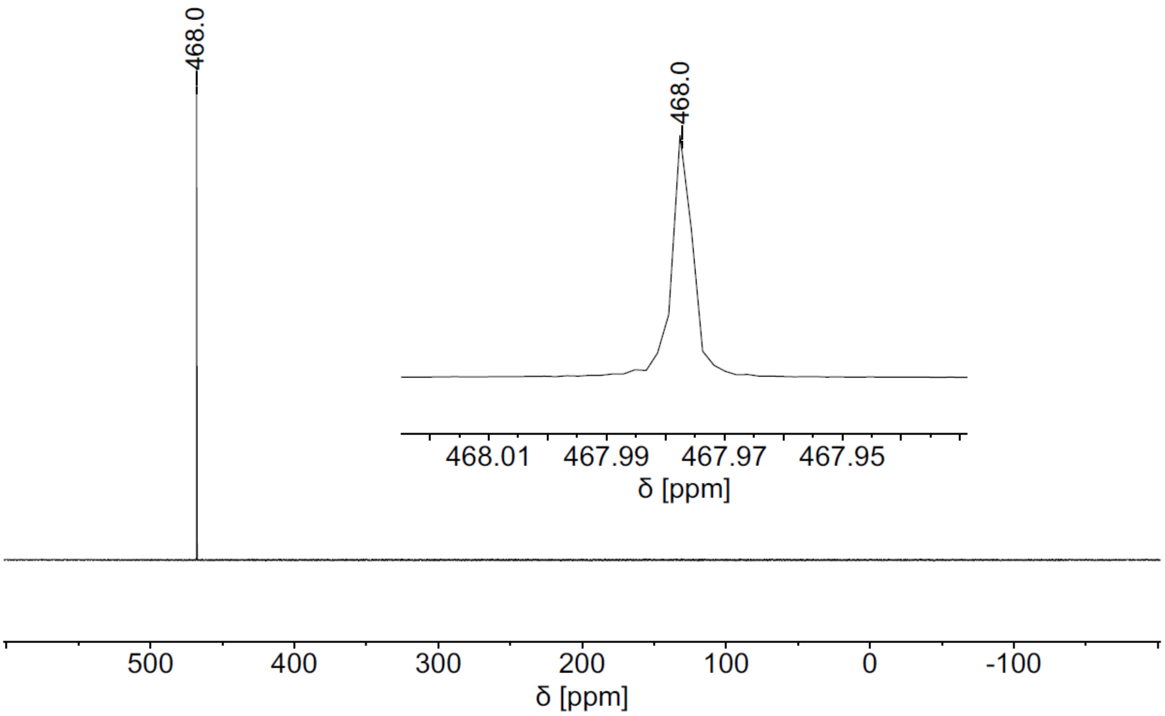

Figure S5. $^{31}P\{^1H\}$ NMR spectrum (thf-d_8) of $[P_5][Na([2.2.2]cryptand)]$ (**2a**).

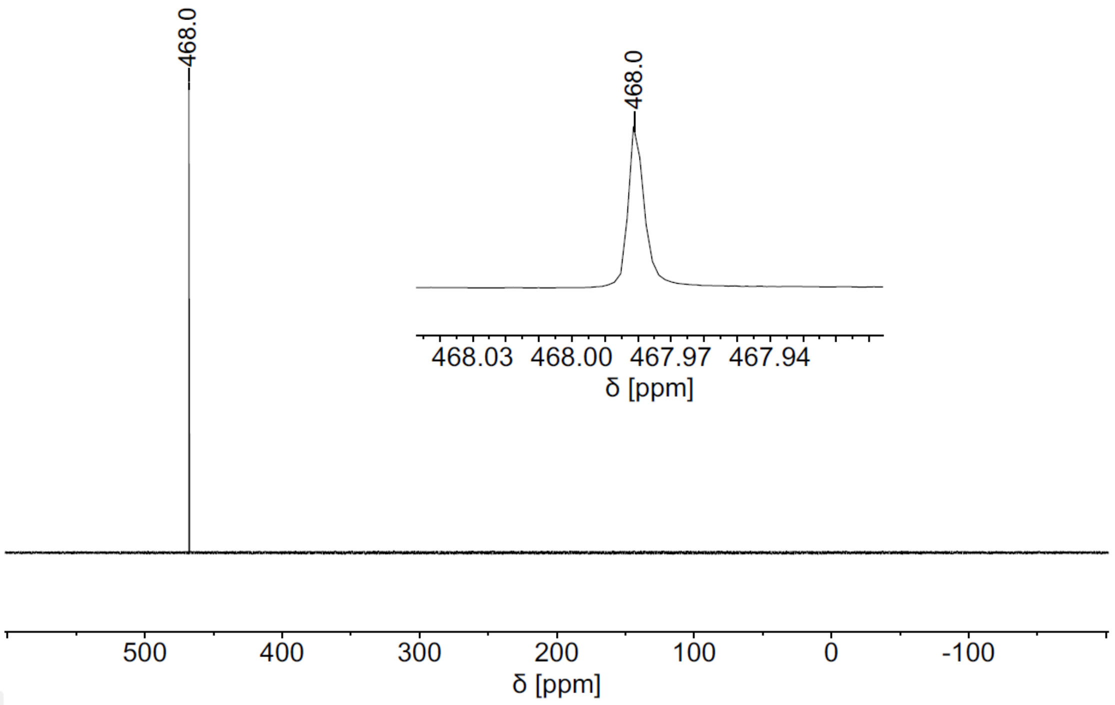

Figure S6. ^{1}H NMR spectrum (thf-d_8) of $[P_5][Na([2.2.2]cryptand)]$ (**2a**).

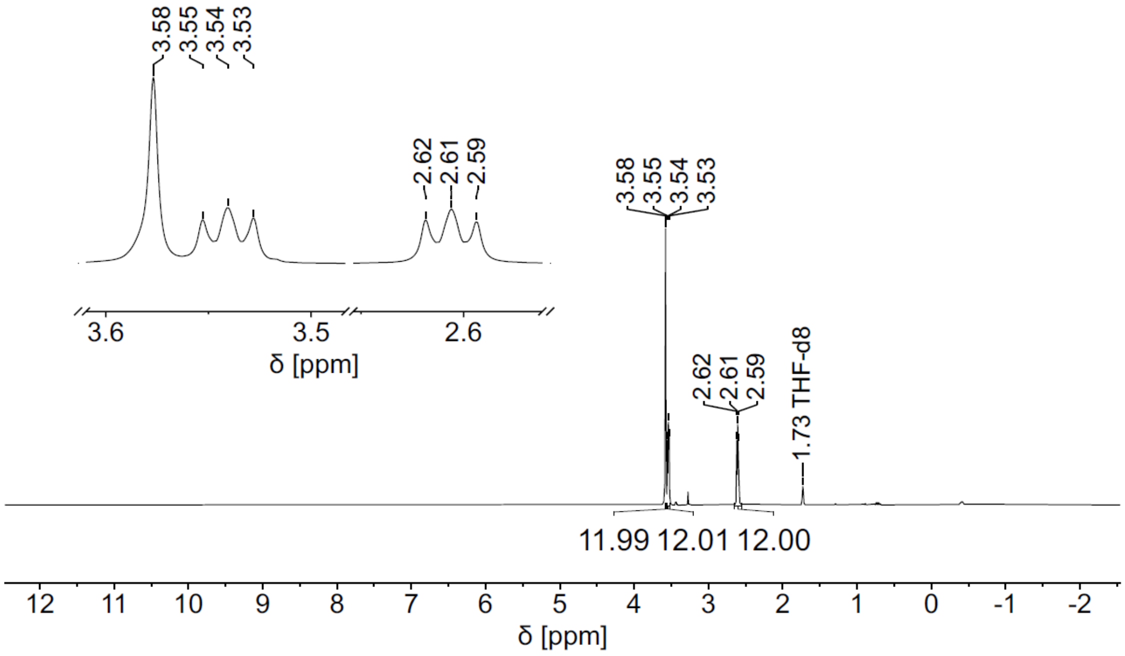

Figure S7. $^{13}C\{^{1}H\}$ NMR spectrum (thf-d_8) of $[P_5][Na([2.2.2]cryptand)]$ (**2a**).

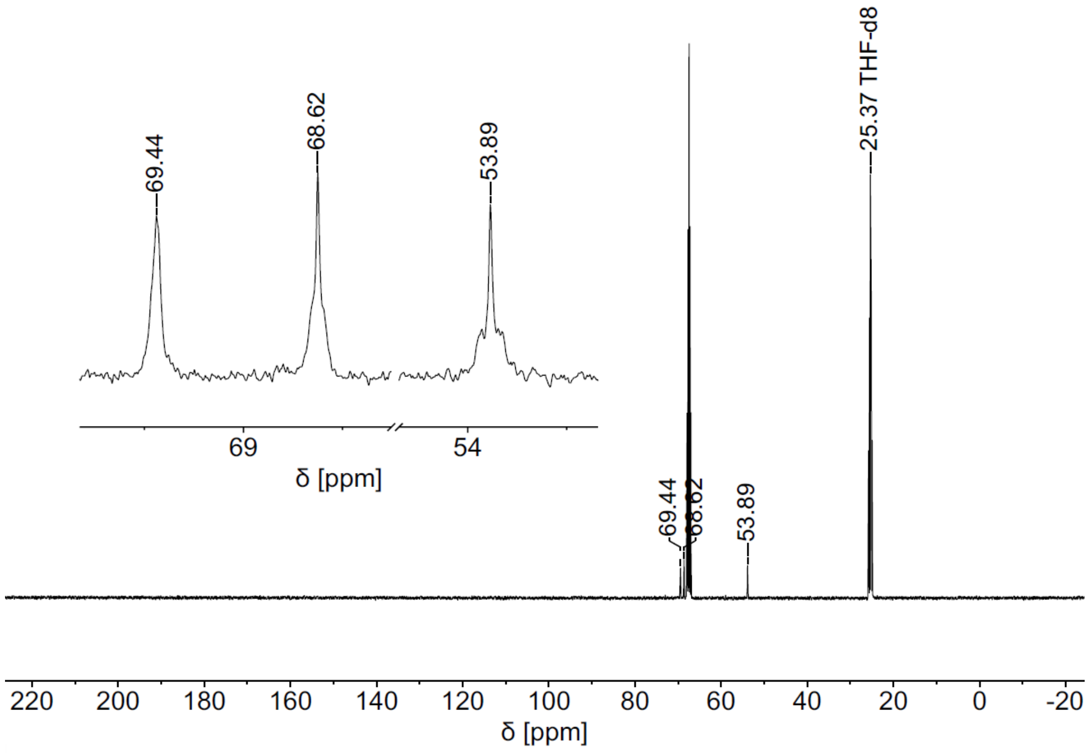

Figure S8. Experimental UV/VIS spectrum of [P_5][Na([2.2.2]cryptand)] (**2a**) recorded in THF solution measured at room temperature.

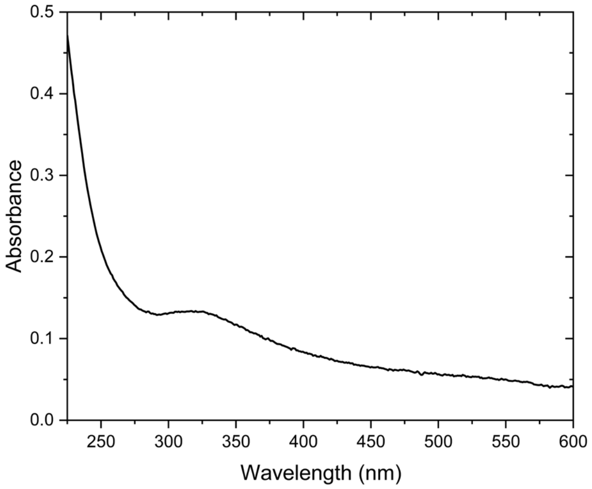

Figure S9. Experimental Raman spectrum of [P_5][Na([2.2.2]cryptand)] (**2a**) recorded at room temperature.

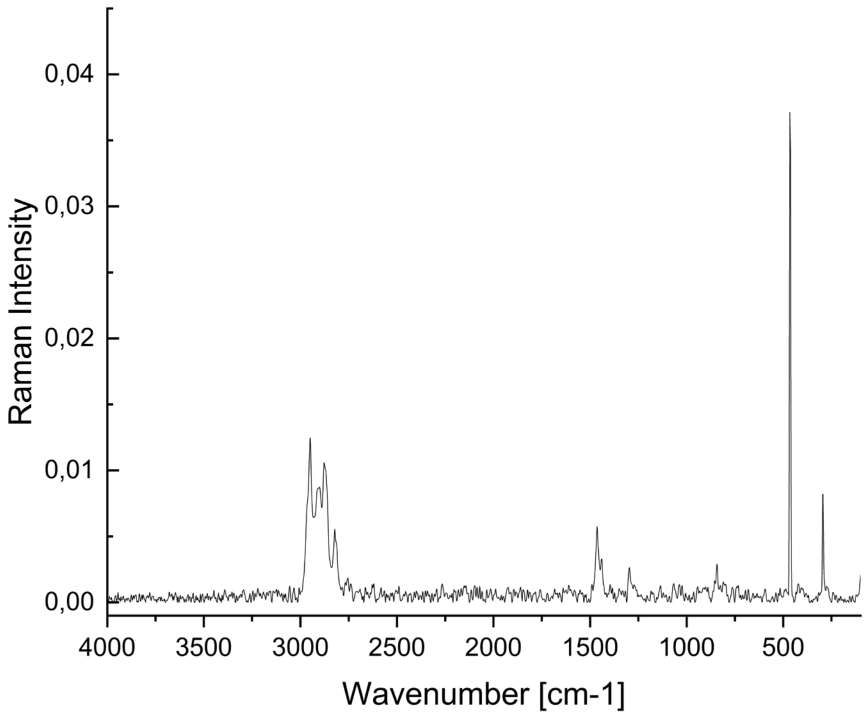

Figure S10. Experimental infra-red (IR) spectrum of [P_5][Na([2.2.2]cryptand)] (**2a**) recorded at room temperature.

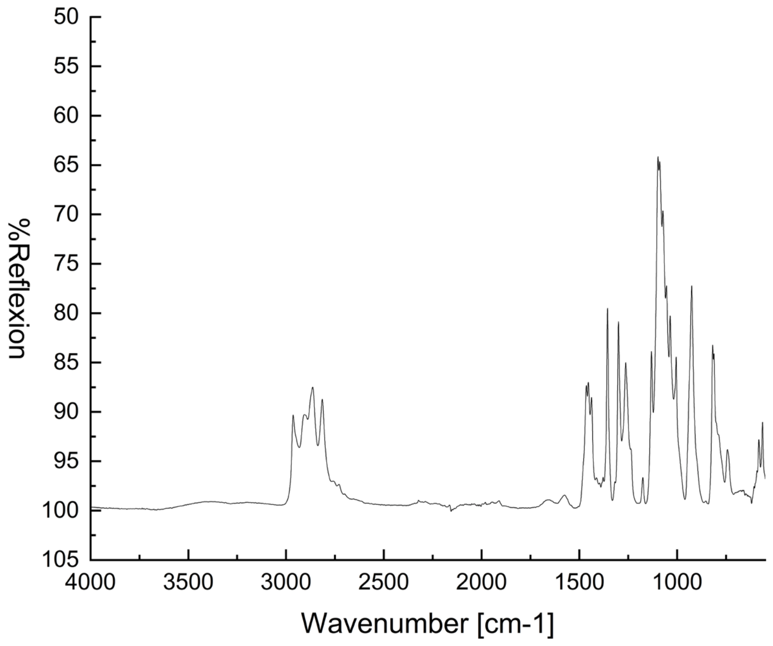

Figure S11. ESI⁻ MS spectrum of [P_5][Na([2.2.2]cryptand)] (**2a**).

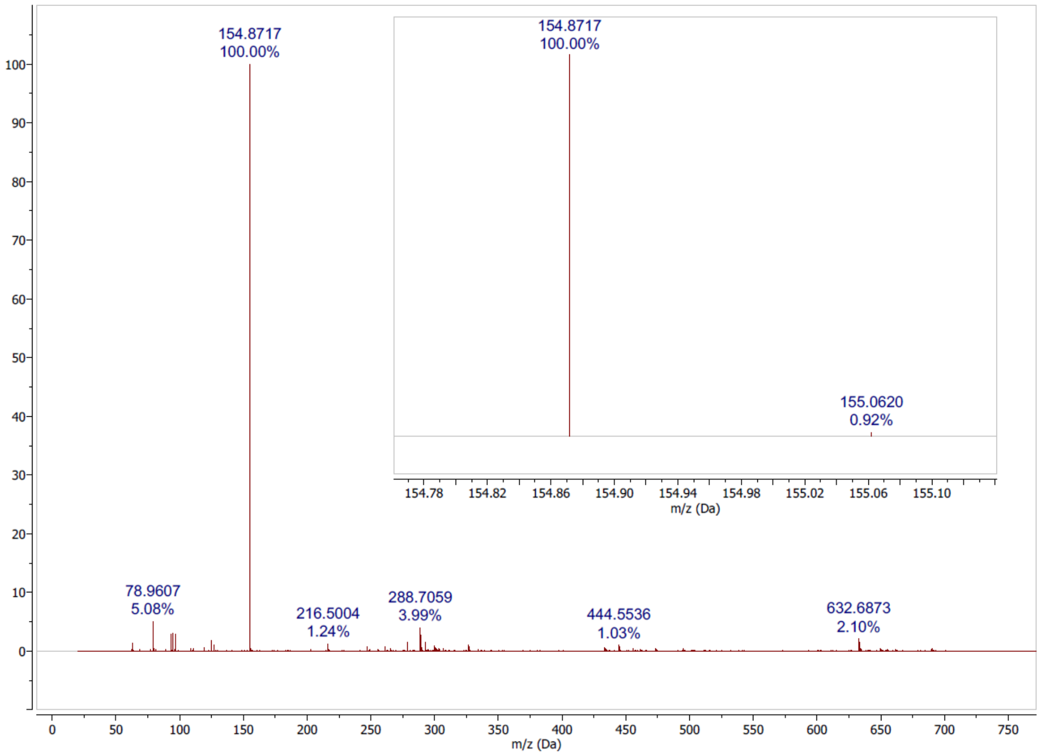

Figure S12. ESI+ MS spectrum of $[P_5][Na([2.2.2]cryptand)]$ (**2a**).

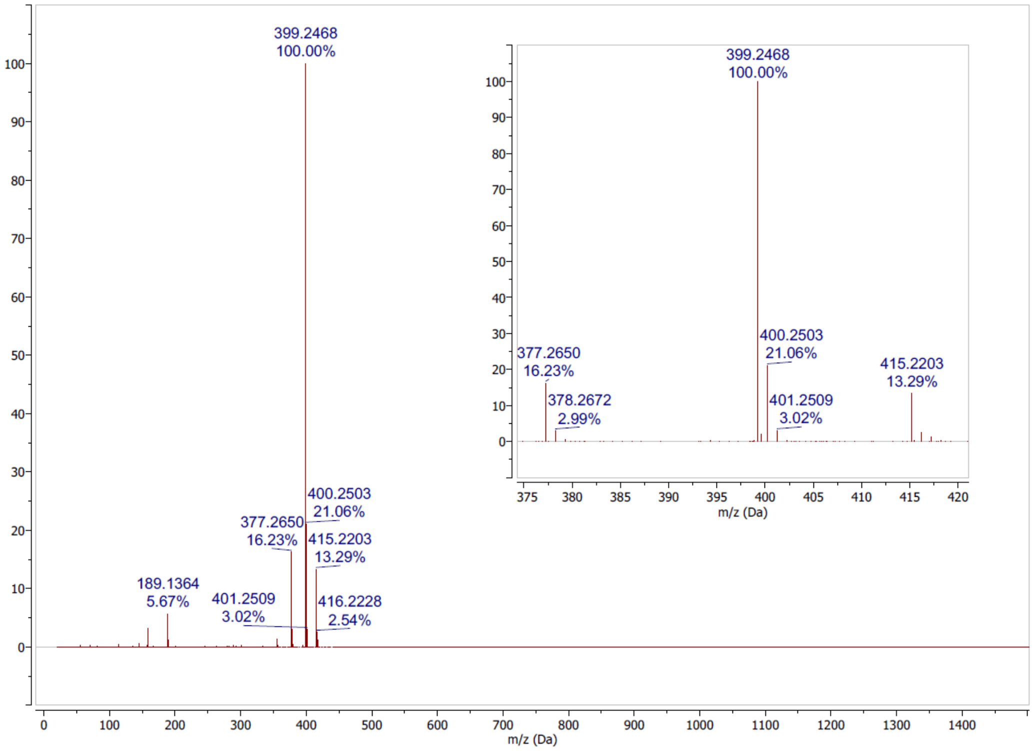

Figure S13. ^{31}P solid-state NMR spectrum of $[P_5][Na([2.2.2]cryptand)]$ (**2a**) with a spinning-rate of 10 kHz.

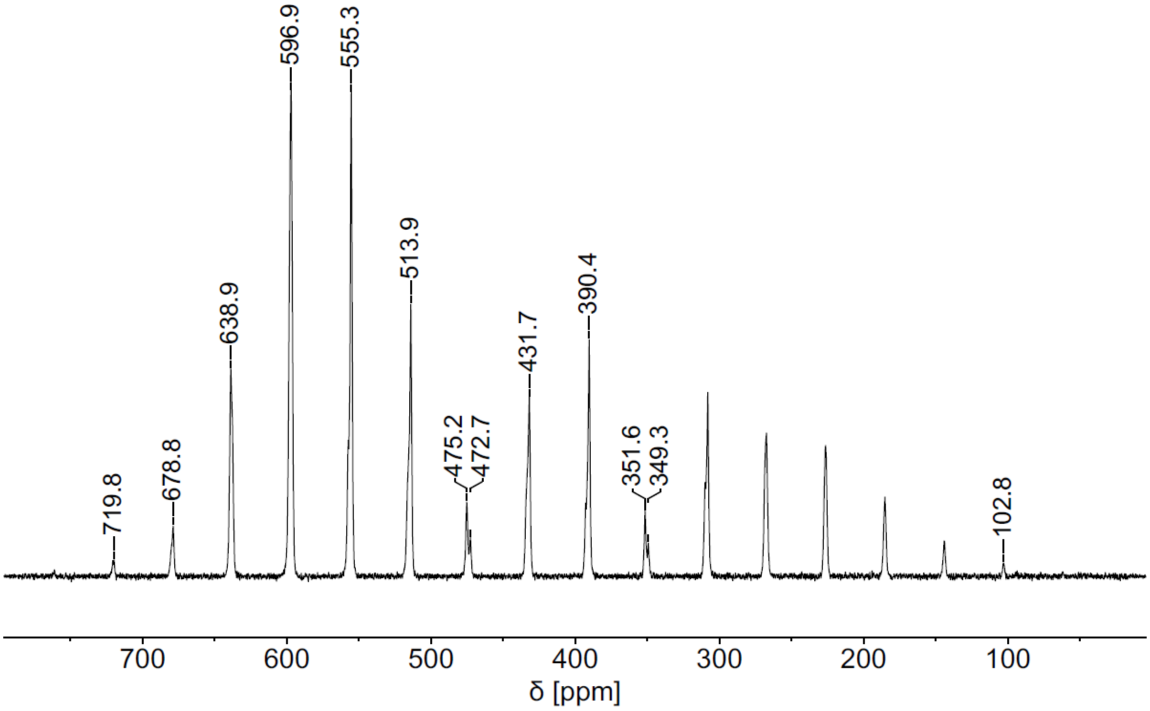

Figure S14. Superimposed ^{31}P solid-state NMR spectrum of $[P_5][Na([2.2.2]cryptand)]$ (**2a**) with a spinning-rates of 10 kHz (blue) and 7 kHz (red).

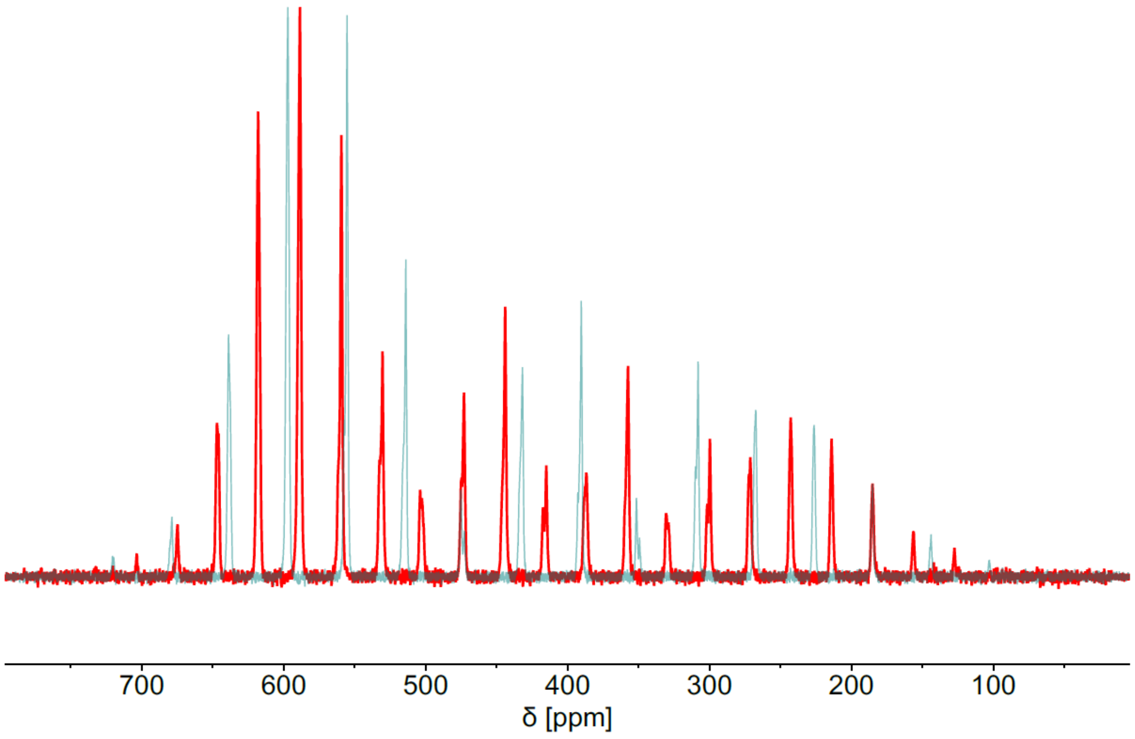

Figure S15. Stacked ^{31}P solid-state NMR spectrum of $[P_5][Na([2.2.2]cryptand)]$ (**2a**) with a spinning-rates of 10 kHz (blue) and 7 kHz (red).

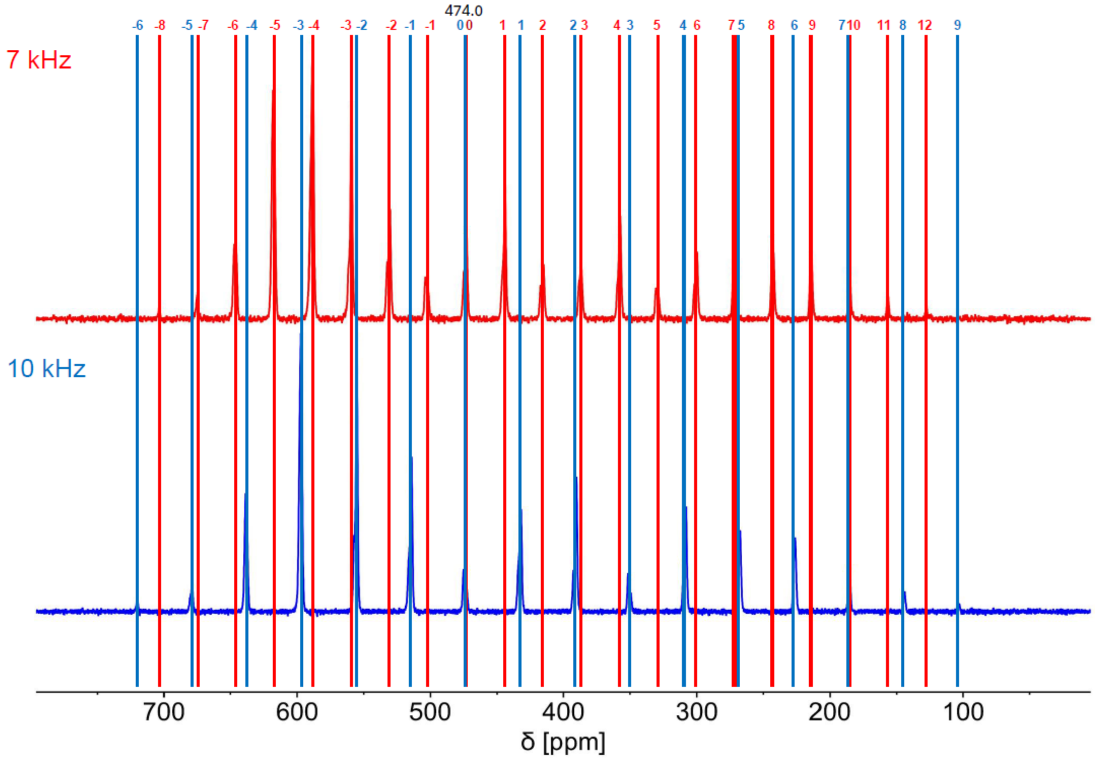

Figure S16. ^{31}P solid-state NMR (toss cogwheel sideband suppression) spectrum of $[P_5][Na([2.2.2]cryptand)]$ (**2a**) with a spinning-rate of 10 kHz.

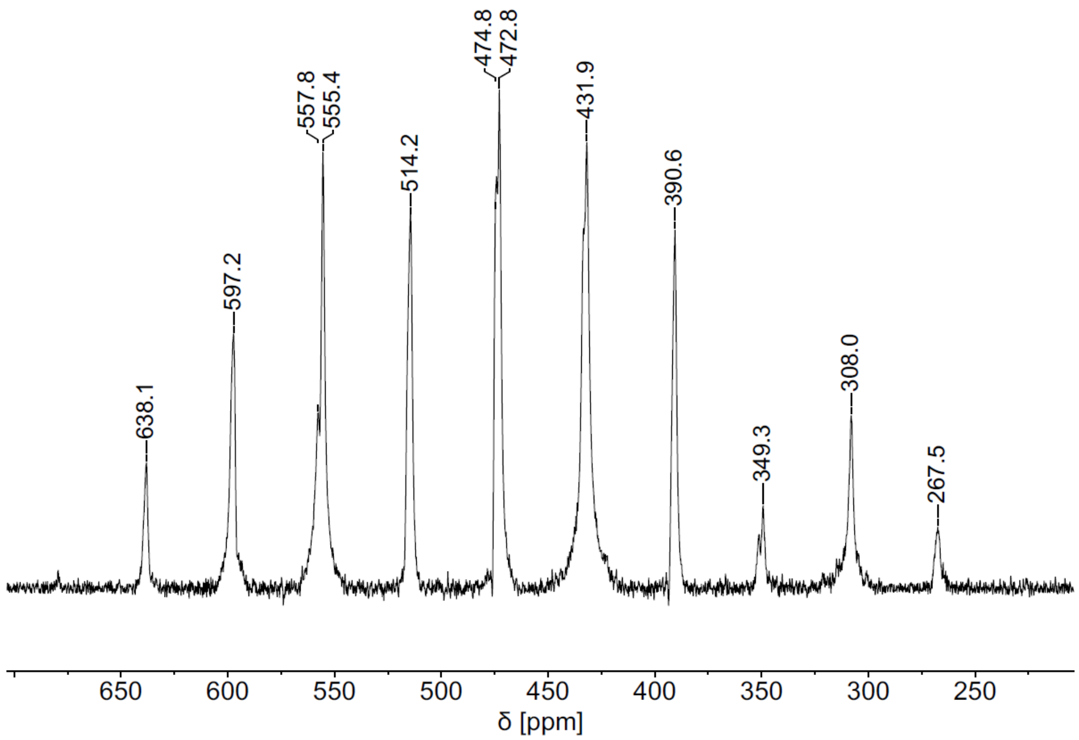

Figure S17. ^{31}P NMR spectrum (CH_2Cl_2) of $[P_5][K([2.2.2]cryptand)]$ (**2b**).

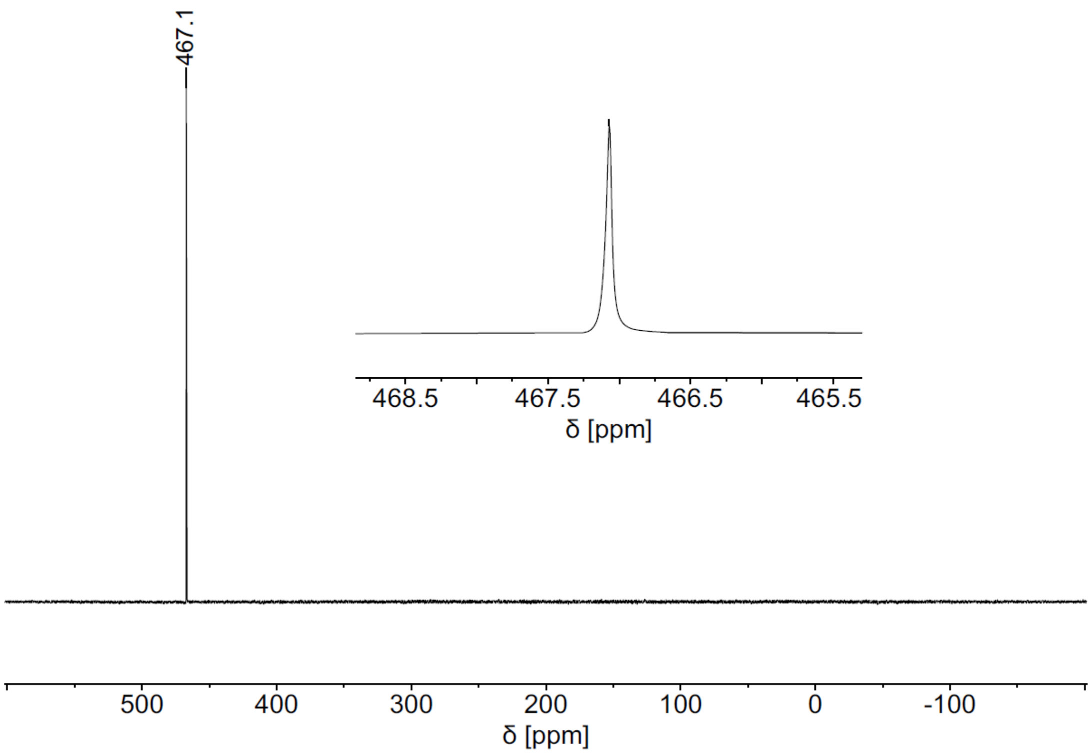

Figure S18. $^{31}P\{^1H\}$ NMR spectrum (CH_2Cl_2) of $[P_5][K([2.2.2]cryptand)]$ (**2b**).

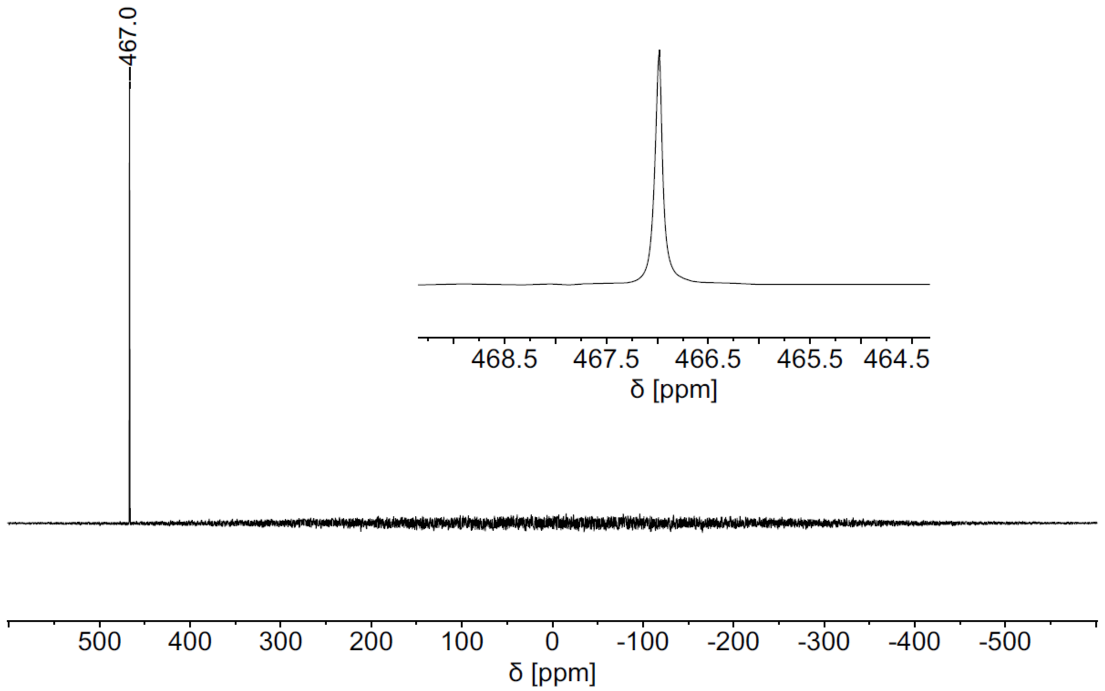

Figure S19. ^1H NMR spectrum (CH_2Cl_2) of $[P_5][K([2.2.2]cryptand)]$ (**2b**).

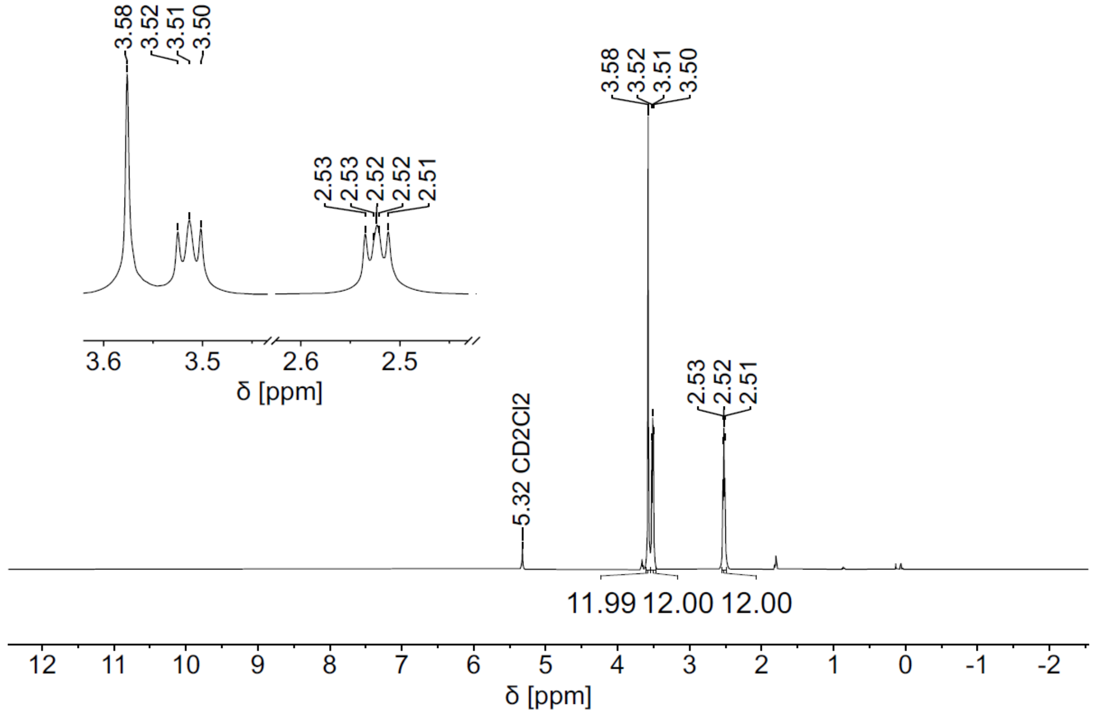

Figure S20. $^{13}C\{^{1}H\}$ NMR spectrum (CH_2Cl_2) of [P_5][K([2.2.2]cryptand)] (**2b**).

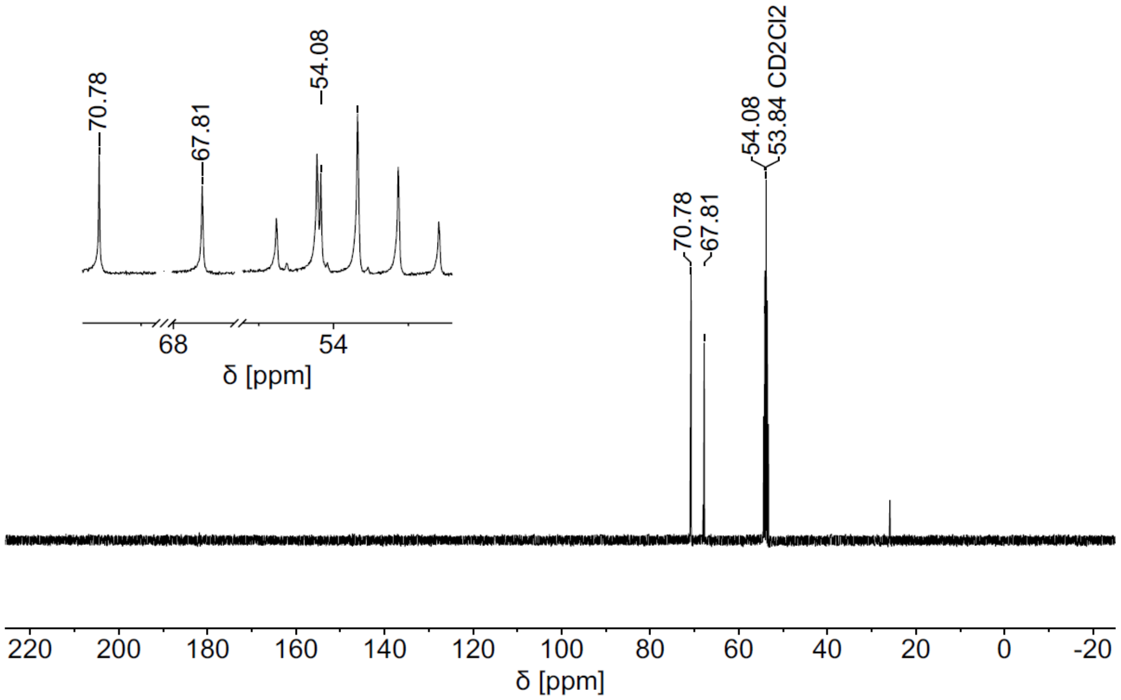

Figure S21. Experimental UV/VIS spectrum of [P_5][K([2.2.2]cryptand)] (**2a**) recorded in THF solution measured at room temperature.

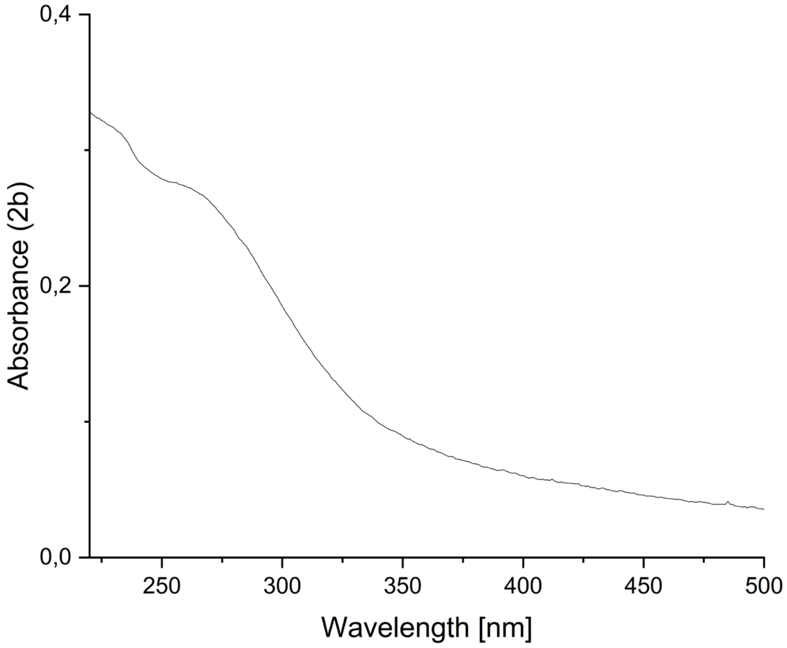

Figure S22. $^{31}P\{^{1}H\}$ NMR spectrum (CH_2Cl_2) of [Cp*Fe(*cyclo*-P_5)].

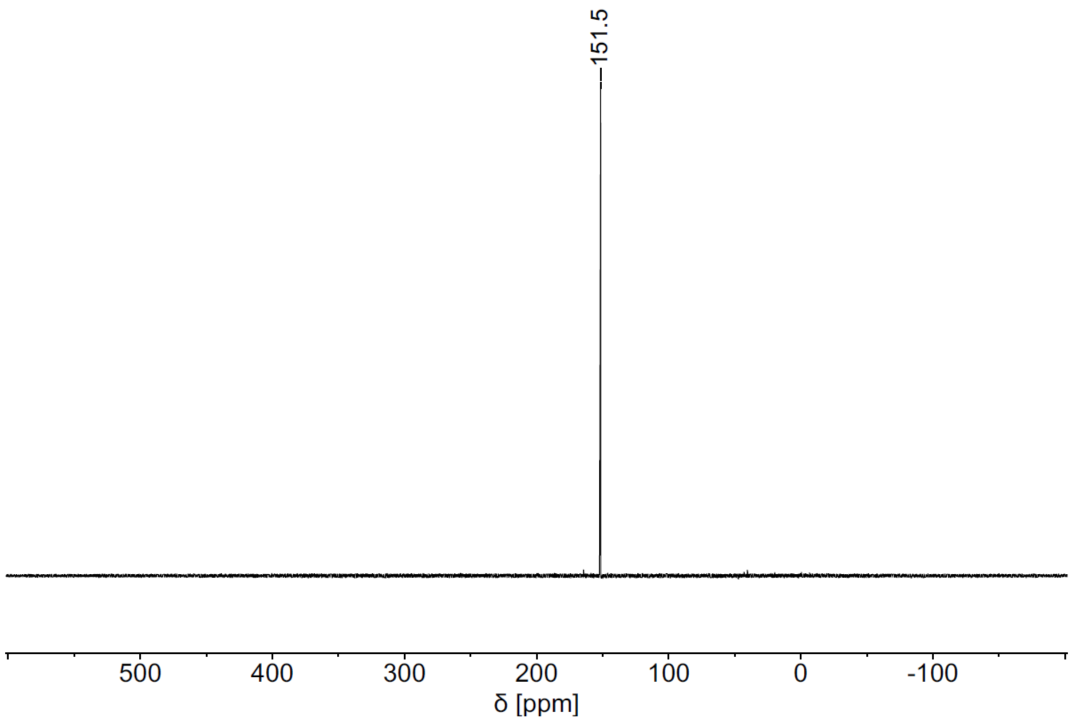

Figure S23. ^{31}P NMR spectrum (CH_2Cl_2) of [Cp*Fe(*cyclo*-P_5)].

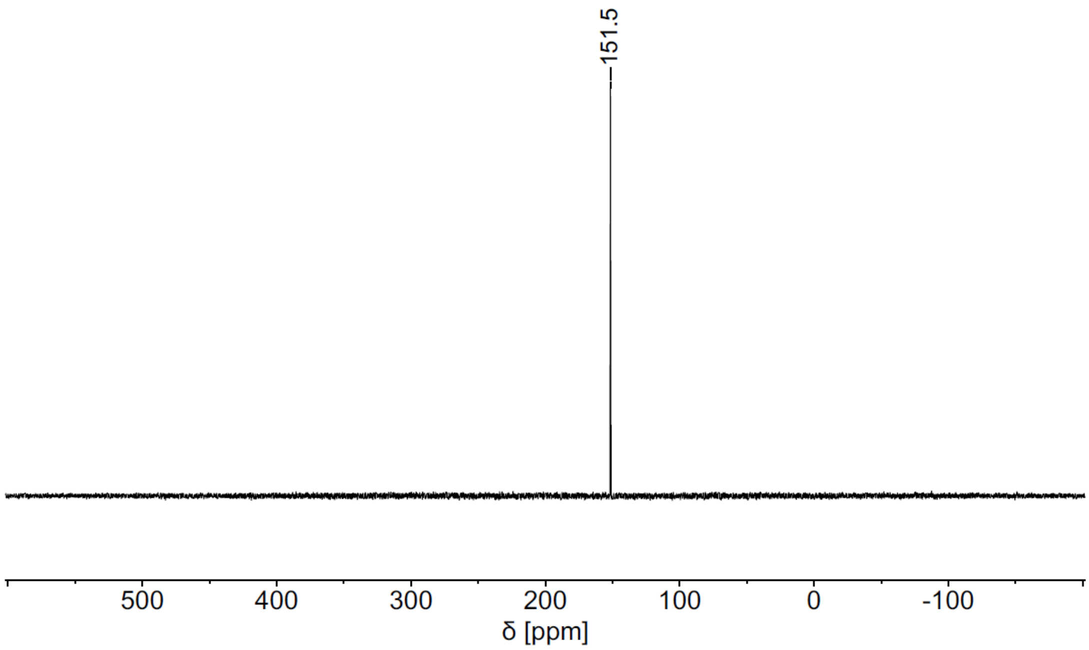

Figure S24. $^{31}P\{^1H\}$ NMR spectrum (thf) of the residue after the filtration of in the synthesis of $[P_5][K([2.2.2]cryptand)]$.

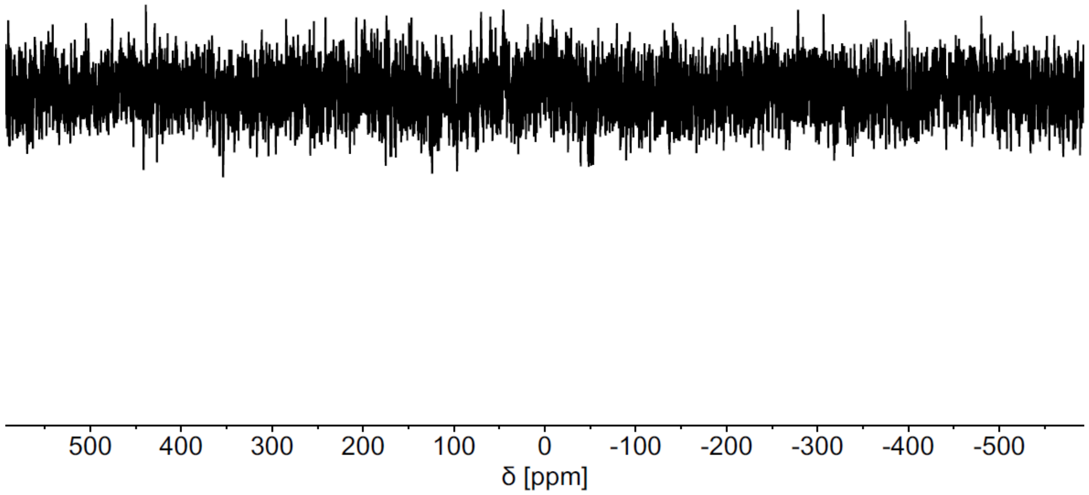

Figure S25. $^{31}P\{^1H\}$ NMR spectrum (thf) of the residue after the filtration of in the synthesis of $[P_5][K([2.2.2]cryptand)]$ after the addition of 0.1 mL H_2O.

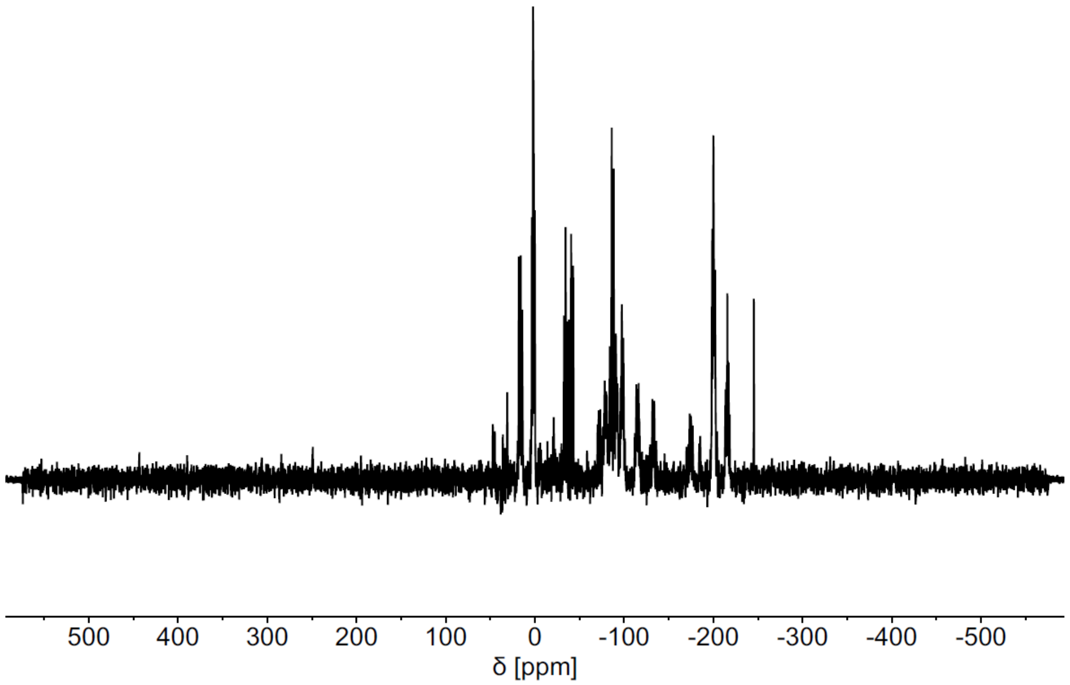

Figure S26. ^{31}P NMR spectrum (thf) of the residue after the filtration of in the synthesis of [P_5][K([2.2.2]cryptand)] after the addition of 0.1 mL H_2O.

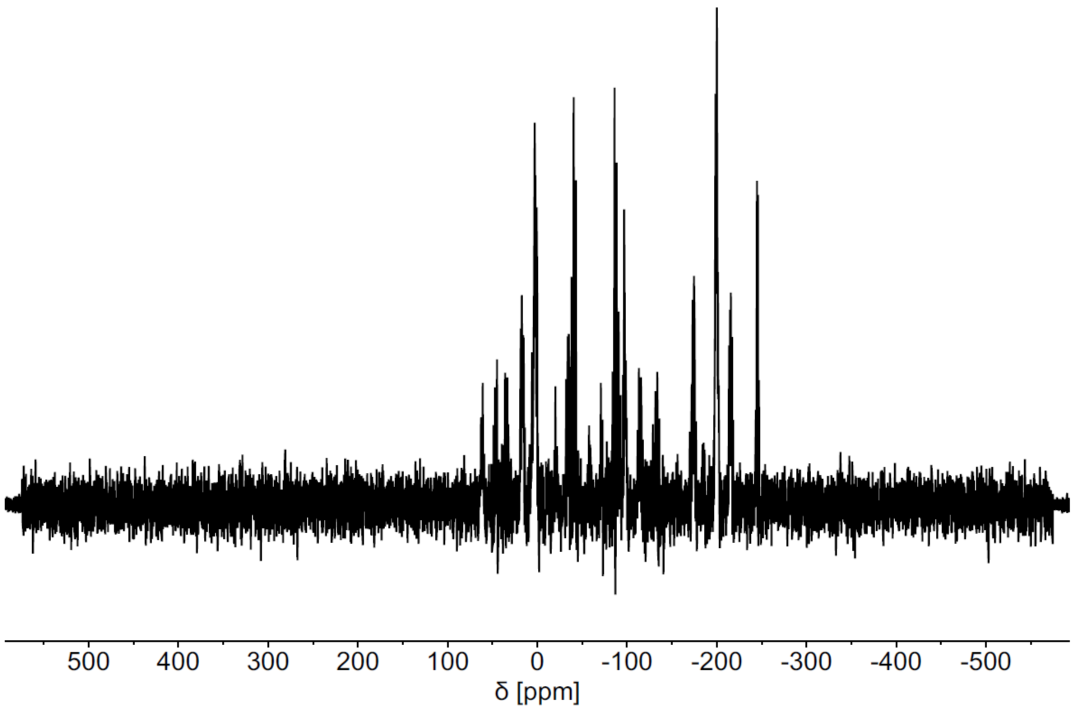

Figure S27. Experimental Raman spectrum of the residue after the filtration of in the synthesis of [P_5][K([2.2.2]cryptand)] recorded at room temperature.

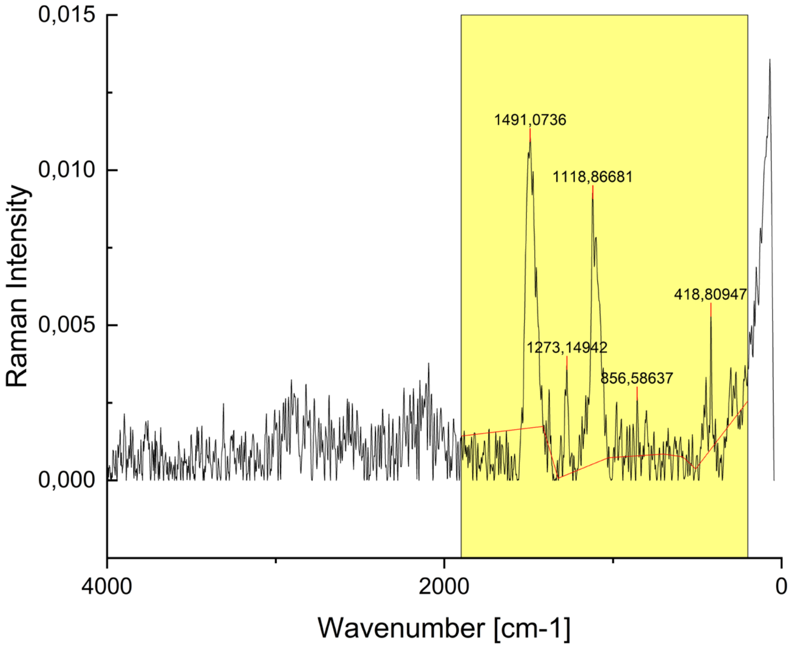

Figure S28. Experimental Raman spectrum of [P_7][K_3(dme)$_{0.12}$] (**1b**) recorded at room temperature.

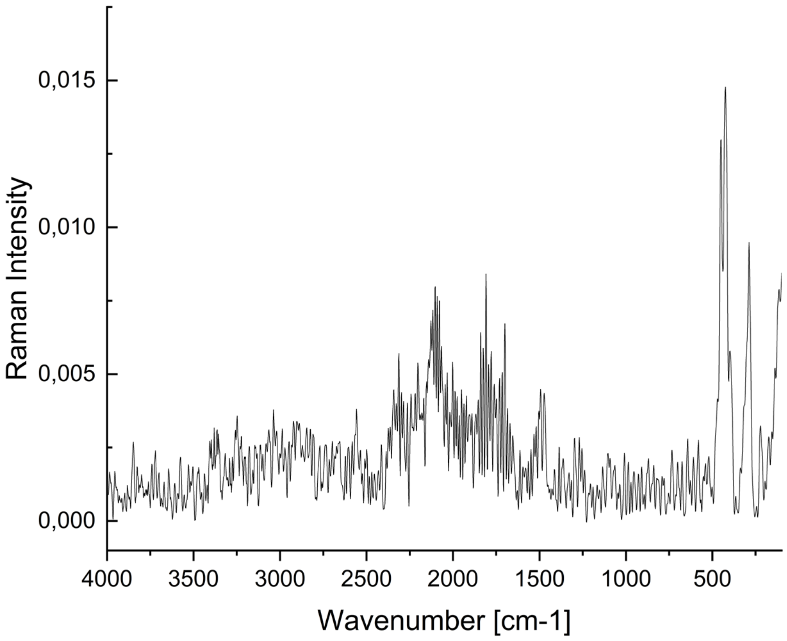

Figure S29. Experimental Raman spectrum of [2.2.2]cryptand recorded at room temperature.

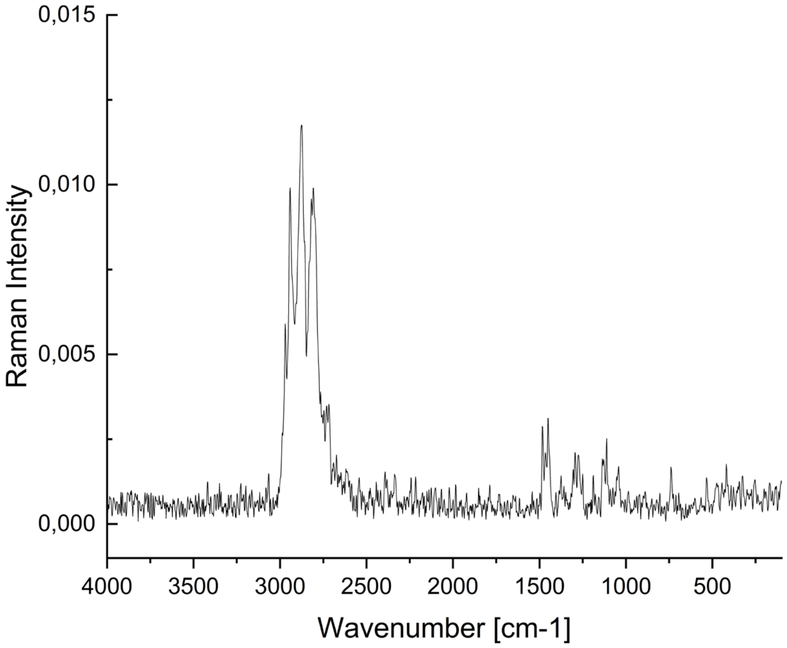

Figure S30. ^{31}P solid-state NMR spectrum of $[P_7][Na_3(dme)_{0.64}]$ (**1a**) with a spinning-rate of 10 kHz.

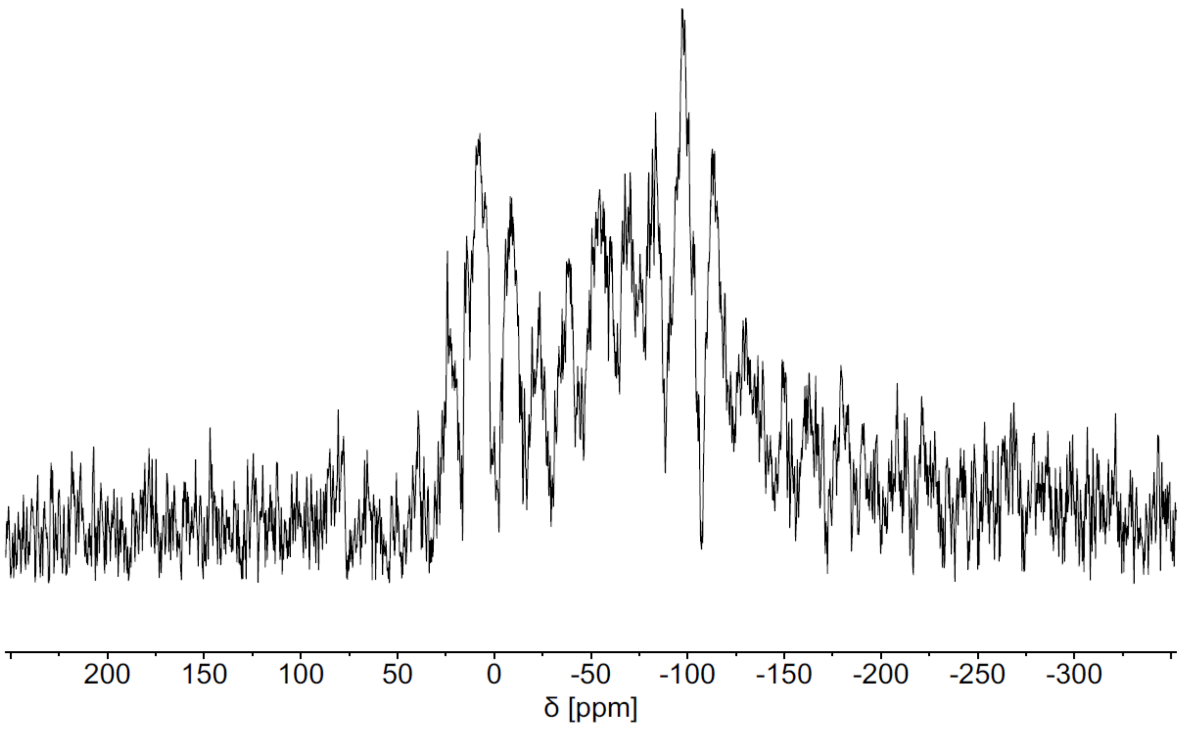

Part 4: Computational and ^{31}P solid-state NMR simulation details

1. General remarks and geometry optimization

Calculations were performed using *Gaussian16* program.[51] *Gaussian16* formatted checkpoint files were used to perform Kohn-Sham MO visualization in *Multiwfn 3.6*.[52] Please note that all computations were carried out for single, isolated molecules in the gas phase (ideal gas approximation). There may well be significant differences between gas phase and condensed phase.

Structures of cyclopentadienide anion, PH_3 and *cyclo*-P_5^- were optimized at the PBE0[53] level of density functional theory in combination with the def2-TZVP[54] basis set and GD3BJ[55,56] empirical dispersion (Notation: PBE0-D3/def2-TZVP). According to vibrational analysis (PBE0-D3/def2-TZVP) optimized structures were characterized as energy minima on the potential energy surface with no imaginary frequencies. For plotting the calculated Raman spectrum of *cyclo*-P_5^- the scaling factor of 0.9591 was used.[57]

Optimized coordinates for *cyclo*-P_5^-:

P	1.2491880000	-1.2649690000	0.0000430000
P	1.5891030000	0.7969600000	0.0000390000
P	-0.2670370000	1.7577210000	-0.0001070000
P	-1.7540210000	0.2891670000	0.0001340000
P	-0.8172340000	-1.5788790000	-0.0001090000

Optimized coordinates for Cp^-:

C	1.1452977398	-0.3493009591	-0.0000380286
C	0.6861800643	0.9811330312	0.0000619714
C	-0.7212685772	0.9556554559	-0.0000870286
C	-1.1320416285	-0.3905622057	-0.0001920286
C	0.0217583487	-1.1968146492	0.0000929714
H	-1.3753745230	1.8221386783	0.0002069714
H	1.3084619912	1.8707441235	0.0000249714
H	2.1837503837	-0.6661176863	0.0001629714
H	0.0416536667	-2.2822662144	-0.0002480286
H	-2.1580471672	-0.7451629497	0.0008259714

2. ^{31}P NMR shift calculations

^{31}P NMR chemical shift of *cyclo*-P_5^- in gaseous phase was calculated using GIAO method[58-62] at PBE0-D3/def2-TZVP level of theory. The calculated absolute shifts ($\delta_{calc,X}$) were referenced to the experimental absolute shift of 85% H_3PO_4 in the gas phase ($\delta_{ref,1}$ = 328.35 ppm)[63] using PH_3 ($\delta_{ref,2}$ = 594.45 ppm)[64] as a secondary standard:

$$\delta_{calc,P5-} = (\delta_{ref,1} - \delta_{ref,2}) - (\delta_{calc,X} - \delta_{calc,PH3}) = \delta_{calc,PH3} - \delta_{calc,X} - 266.1 \text{ ppm}$$

At the PBE0-D3/def2-TZVP level of theory (gas phase, GIAO method), $\delta_{calc,PH3}$ amounts to +570.84 ppm. Calculated ^{31}P NMR chemical shift of cyclo-P_5^-: +516 ppm**.**

3. ^{31}P solid-state NMR simulations

Processing the raw data in preparation for fitting and the fitting of CSA-parameters itself were conducted using the Python program ssNake (V.1.5). [65] The spectra were fit simultaneously by linking the corresponding CSA parameters. The Integral and the parameters for Gaussian and Lorentzian linewidths remained independent in each case.

4. TD-DFT calcuations

TD-DFT calculations were performed at the PBE0-D3/def2-TZVP level of theory. For TD-DFT calculations 15 excited states were considered as well as the SCRF approach for THF as a solvent.

Excitation energies and oscillator strengths:

```
 Excited State   1:      Singlet-A      3.3680 eV  368.12 nm  f=0.0000  <S**2>=0.000
      37 -> 39         0.41022
      37 -> 40        -0.28269
      38 -> 39         0.28564
      38 -> 40         0.41094
 This state for optimization and/or second-order correction.
 Total Energy, E(TD-HF/TD-DFT) =  -1706.26631434
 Copying the excited state density for this state as the 1-particle RhoCI density.

 Excited State   2:      Singlet-A      3.3681 eV  368.12 nm  f=0.0000  <S**2>=0.000
      37 -> 39        -0.28379
      37 -> 40        -0.40817
      38 -> 39         0.41298
      38 -> 40        -0.28454

 Excited State   3:      Singlet-A      3.3924 eV  365.48 nm  f=0.0000  <S**2>=0.000
      37 -> 39        -0.21350
      37 -> 40         0.45415
      38 -> 39         0.44914
      38 -> 40         0.21335
```

```
Excited State   4:      Singlet-A      3.3924 eV  365.47 nm  f=0.0000  <S**2>=0.000
     37 -> 39         0.45218
     37 -> 40         0.21472
     38 -> 39         0.21212
     38 -> 40        -0.45112

Excited State   5:      Singlet-A      3.7355 eV  331.91 nm  f=0.0000  <S**2>=0.000
     35 -> 39        -0.11158
     35 -> 40        -0.48231
     36 -> 39         0.48327
     36 -> 40        -0.11194

Excited State   6:      Singlet-A      3.7357 eV  331.89 nm  f=0.0000  <S**2>=0.000
     35 -> 39         0.48240
     35 -> 40        -0.11158
     36 -> 39         0.11193
     36 -> 40         0.48317

Excited State   7:      Singlet-A      4.1576 eV  298.21 nm  f=0.1737  <S**2>=0.000
     35 -> 39         0.41540
     35 -> 40        -0.24436
     36 -> 39        -0.24399
     36 -> 40        -0.41461

Excited State   8:      Singlet-A      4.1581 eV  298.17 nm  f=0.1735  <S**2>=0.000
     35 -> 39         0.24433
     35 -> 40         0.41548
     36 -> 39         0.41449
     36 -> 40        -0.24400

Excited State   9:      Singlet-A      4.8417 eV  256.08 nm  f=0.0000  <S**2>=0.000
     33 -> 39        -0.38901
     33 -> 40        -0.30307
     34 -> 39        -0.30345
     34 -> 40         0.38914

Excited State  10:      Singlet-A      4.9780 eV  249.06 nm  f=0.0282  <S**2>=0.000
     33 -> 39        -0.30537
     33 -> 40         0.39128
     34 -> 39         0.39245
     34 -> 40         0.30548

Excited State  11:      Singlet-A      5.0825 eV  243.94 nm  f=0.0000  <S**2>=0.000
     33 -> 39         0.32311
     33 -> 40         0.37757
     34 -> 39        -0.37671
     34 -> 40         0.32333

Excited State  12:      Singlet-A      5.0826 eV  243.94 nm  f=0.0000  <S**2>=0.000
     33 -> 39         0.37734
     33 -> 40        -0.32359
     34 -> 39         0.32285
     34 -> 40         0.37694

Excited State  13:      Singlet-A      5.2437 eV  236.45 nm  f=0.0000  <S**2>=0.000
     35 -> 41         0.14354
     35 -> 42         0.46646
     36 -> 41         0.47827
     36 -> 42        -0.14298

Excited State  14:      Singlet-A      5.2848 eV  234.60 nm  f=0.0000  <S**2>=0.000
```

```
    37 -> 42         0.49341
    38 -> 41         0.50403

 Excited State  15:      Singlet-A      5.3408 eV  232.15 nm  f=0.0006  <S**2>=0.000
    35 -> 41         0.49853
    35 -> 42        -0.12031
    36 -> 41        -0.16686
    36 -> 42        -0.45014
 SavETr:  write IOETrn=   770 NScale= 10 NData=  16 NLR=1 NState=   15 LETran=     280.
```

For plotting the calculated spectra full width at half maximum (FWHM) parameter was set at 0.66 eV.

Figure S31. Experimental (red) and calculated (black) UV-Vis spectra for [P_5][M([2.2.2]cryptand)] (M = Na (**2a**), K (**2b**)) recorded in thf solution measured at room temperature:

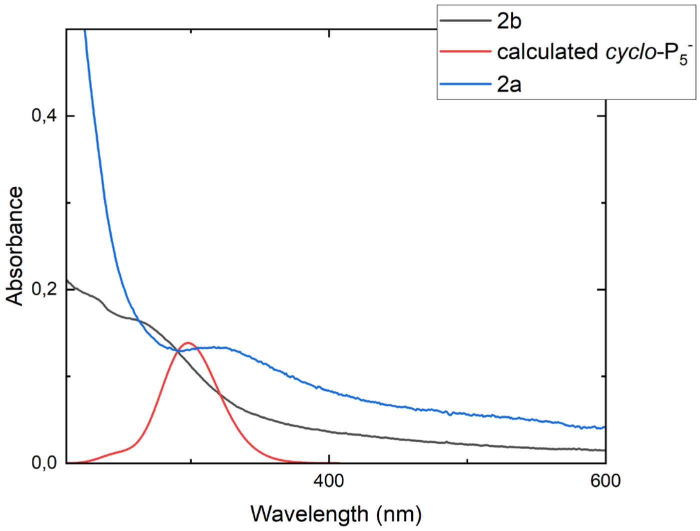

The nature of the absorption band around 300 nm (excitation states 7 and 8) was elucidated based on the electronic excitation analysis performed in *Multiwfn* program.

Charge density difference plots that correspond to excitation states 7 and 8 reveal $\pi \rightarrow \pi^*$ nature of the absorption band around 300 nm.

Figure S32. Charge density difference plot of for cyclo-P_5^- corresponding to excitation states 7 and 8 (isosurface value: 0.002. red region is electron donating and blue region is electron accepting):

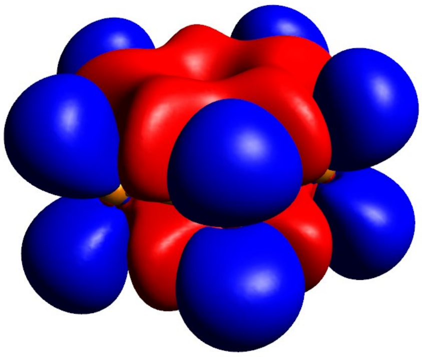

References

(as cited in manuscript)

[5] M. Baudler, S. Akpapoglou, D. Ouzounis, F. Wasgestian, B. Meinigke, H. Budzikiewicz, H. Münster, *Angew. Chem.,Int. Ed. Engl.* **1988**, *27*, 280–281.

[15] O. J. Scherer, J. Schwalb, G. Wolmershäuser, W. Kaim, R. Gross, *Angew. Chem. Int. Ed. Engl.* **1986**, 25, 363–364.

[35] M. Cicač-Hudi, J. Bender, S. H. Schlindwein, M. Bispinghoff, M. Nieger, H. Grützmacher, D. Gudat, *Eur. J. Inorg. Chem.* **2016**, *2016*, 649–658.

[41] MestReNova 14.1.0-24037, Mestrelab Reasearch S.L.: **2019**.

[42] Bruker, *APEX III.* Bruker AXS Inc., Madison, Wisconsin, USA: **2019**.

[43] Bruker, *Apex 4.* Bruker AXS Inc.; Madison, WI, USA: **2021**.

[44] Sheldrick, G. M. *SADABS*; *University of Göttingen: Germany* **1996**.

[45] Coppens, P. The Evaluation of Absorption and Extinction in Single-Crystal Structure Analysis. *Crystallographic Computing, Copenhagen, Muksgaard* **1979**.

[46] O. V. Dolomanov, L. J. Bourhis, R. J. Gildea, J. a. K. Howard, H. Puschmann, *J. Appl. Cryst.* **2009**, *42*, 339–341.

[47] Sheldrick, G. M. A short history of SHELX. *Acta Crystallogr., Sect. A: Found. Crystallogr.* **2008**, *A64*, 112.

[48] Sheldrick, G. M. Crystal structure refinement with SHELXL. *Acta Crystallogr., Sect. C: Struct. Chem.* **2015**, *C71*, 3.

[49] The facility “CheckCIF,” can be found at http://checkcif.iucr.org.

[50] *Diamond - Crystal and Molecular Structure Visualization; Crystal Impact* - Dr. H. Putz & Dr. K. Brandenburg GbR: Bonn, Germany. **2019**.

[51] M. J. Frisch, G. W. Trucks, H. B. Schlegel, G. E. Scuseria, M. A. Robb, J. R. Cheeseman, G. Scalmani, V. Barone, G. A. Petersson, H. Nakatsuji, X. Li, M. Caricato, A. V. Marenich, J. Bloino, B. G. Janesko, R. Gomperts, B. Mennucci, H. P. Hratchian, J. V. Ortiz, A. F. Izmaylov, J. L. Sonnenberg, D. Williams-Young, F. Ding, F. Lipparini, F. Egidi, J. Goings, B. Peng, A. Petrone, T. Henderson, D. Ranasinghe, V. G.

Zakrzewski, J. Gao, N. Rega, G. Zheng, W. Liang, M. Hada, M. Ehara, K. Toyota, R. Fukuda, J. Hasegawa, M. Ishida, T. Nakajima, Y. Honda, O. Kitao, H. Nakai, T. Vreven, K. Throssell, J. A. Montgomery, Jr., J. E. Peralta, F. Ogliaro, M. J. Bearpark, J. J. Heyd, E. N. Brothers, K. N. Kudin, V. N. Staroverov, T. A. Keith, R. Kobayashi, J. Normand, K. Raghavachari, A. P. Rendell, J. C. Burant, S. S. Iyengar, J. Tomasi, M. Cossi, J. M. Millam, M. Klene, C. Adamo, R. S49 Cammi, J. W. Ochterski, R. L. Martin, K. Morokuma, O. Farkas, J. B. Foresman, D. J. Fox, *Gaussian*, Inc., Wallingford CT, **2016**.

[52] T. Lu, F. Chen, *J. Comput. Chem.*, **2012**, *33*, 580–592.

[53] C. Adamo, V. Barone, *The J. Chem. Phys.* **1999**, *110*, 6158–6170.

[54] F. Weigend, R. Ahlrichs, *Phys. Chem. Chem. Phys.* **2005**, *7*, 3297–3305

[55] S. Grimme, J. Antony, S. Ehrlich, H. Krieg, *J. Chem. Phys.* **2010**, *132*, 154104–154104.

[56] S. Grimme, S. Ehrlich, L. Goerigk, *J. Comput. Chem.* **2011**, *32*, 1456–1465.

[57] D. S. Tikhonov, I. Gordiy, D. A. Iakovlev, A. A. Gorislav, M. A. Kalinin, S. A. Nikolenko, K. M. Malaskeevich, K. Yureva, N. A. Matsokin, M. Schnell, *ChemPhysChem* **2024**, *25*, e202400547.

[58] F. London, *J. Phys. Radium* **1937**, *8*, 397–409.

[59] R. McWeeny, *Phys. Rev.* **1962**, *126*, 1028–1034.

[60] R. Ditchfield, *Mol. Phys.* **1974**, *27*, 789–807.

[61] K. Wolinski, J. F. Hinton, P. Pulay, *J. Am. Chem. Soc.* **1990**, *112*, 8251–8260.

[62] J. R. Cheeseman, G. W. Trucks, T. A. Keith, M. J. Frisch, *J. Chem. Phys.* **1996**, *104*, 5497–5509.

[63] C. J. Jameson, A. De Dios, A. K. Jameson, *Chem. Phys. Lett.* **1990**, *167*, 575–582.

[64] C. van Wüllen, *Phys. Chem. Chem. Phys.* **2000**, *2*, 2137–2144.

[65] S.G.J. van Meerten, W.M.J. Franssen, A.P.M. Kentgens, *J. Magn. Reason.* **2019**, *301*, 56–66.
